# TaS_2_, TaSe_2_, and Their Heterogeneous Films as Catalysts for the Hydrogen Evolution Reaction

**DOI:** 10.1021/acscatal.9b03184

**Published:** 2020-02-10

**Authors:** Leyla Najafi, Sebastiano Bellani, Reinier Oropesa-Nuñez, Beatriz Martín-García, Mirko Prato, Lea Pasquale, Jaya-Kumar Panda, Petr Marvan, Zdeněk Sofer, Francesco Bonaccorso

**Affiliations:** † Graphene Labs, Istituto Italiano di Tecnologia, via Morego 30, 16163 Genova, Italy; § BeDimensional Spa, via Albisola 121, 16163 Genova, Italy; ∥ Materials Characterization Facility, Istituto Italiano di Tecnologia, via Morego 30, 16163 Genova, Italy; ⊥ Department of Inorganic Chemistry, University of Chemistry and Technology Prague, Technická 5, 166 28 Prague 6, Czech Republic

**Keywords:** transition-metal dichalcogenides (TMDs), tantalum disulfide (TaS_2_), tantalum diselenide (TaSe_2_), hydrogen evolution reaction (HER), heterogeneous catalysts

## Abstract

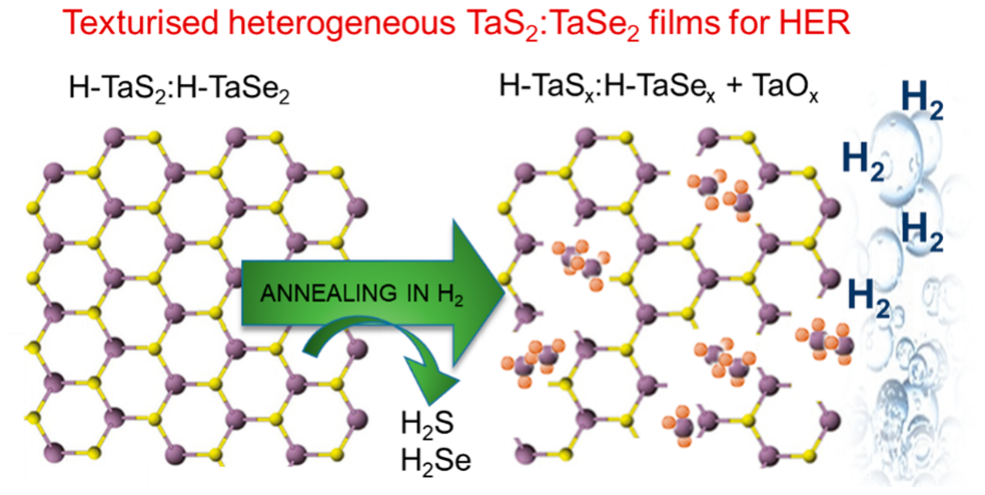

Metallic two-dimensional transition-metal dichalcogenides (TMDs) of the group 5 metals are emerging as catalysts for an efficient hydrogen evolution reaction (HER). The HER activity of the group 5 TMDs originates from the unsaturated chalcogen edges and the highly active surface basal planes, whereas the HER activity of the widely studied group 6 TMDs originates solely from the chalcogen- or metal-unsaturated edges. However, the batch production of such nanomaterials and their scalable processing into high-performance electrocatalysts is still challenging. Herein, we report the liquid-phase exfoliation of the 2H-TaS_2_ crystals by using 2-propanol to produce single/few-layer (1H/2H) flakes, which are afterward deposited as catalytic films. A thermal treatment-aided texturization of the catalytic films is used to increase their porosity, promoting the ion access to the basal planes of the flakes, as well as the number of catalytic edges of the flakes. The hybridization of the H-TaS_2_ flakes and H-TaSe_2_ flakes tunes the Gibbs free energy of the adsorbed atomic hydrogen onto the H-TaS_2_ basal planes to the optimal thermo-neutral value. In 0.5 M H_2_SO_4_, the heterogeneous catalysts exhibit a low overpotential (versus RHE, reversible hydrogen electrode) at the cathodic current of 10 mA cm^–2^ (η_10_) of 120 mV and high mass activity of 314 A g^–1^ at an overpotential of 200 mV. In 1 M KOH, they show a η_10_ of 230 mV and a mass activity of 220 A g^–1^ at an overpotential of 300 mV. Our results provide new insight into the usage of the metallic group 5 TMDs for the HER through scalable material preparation and electrode processing.

## 1 Introduction

Molecular hydrogen (H_2_) has been touted as an ideal energy carrier with high energy density (between 120 and 140 MJ kg^–1^).^1^ In fact, it can be generated by electrochemical water splitting powered by renewable resources,^2^ and its utilization, giving water as a byproduct, is sustainable and environmentally friendly.^3^ To spread the use of H_2_ as energy fuel, i.e., to make feasible the so-called “Hydrogen economy” model,^4,5^ it is crucial to develop efficient electrocatalysts. The latter have to promote the hydrogen evolution reaction (HER) (i.e., 4H_3_O^+^ + 4e^–^ → 4H_2_O + 2H_2_ in acidic media; 4H_2_O + 4e^–^ → 2H_2_ + 4OH^–^ in alkaline media)^6^ accelerating its kinetics.^7^ The most effective electrocatalysts for the HER are expensive and scarce Pt-group elements.^8−10^ Therefore, the upscaling of electrochemical technology for HER is currently inspiring the search for viable catalyst alternatives,^11−14^ including low Pt-content alloys^15−17^ or low-cost transition-metal-based alloys, compounds, and heterostructures.^11−13,18^

**Figure 1:**
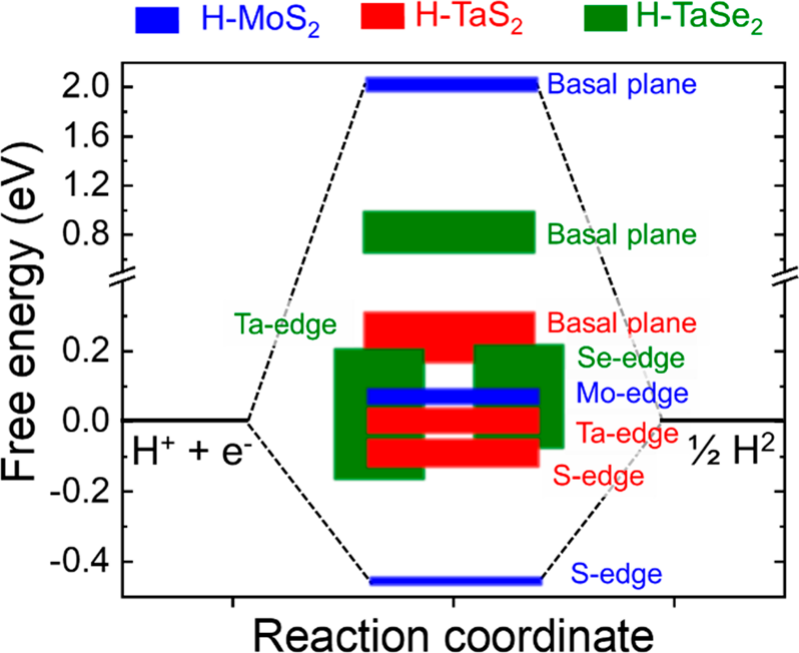
Standard Δ*G*_H_ ranges of different sites of semiconducting H–MoS_2_, metallic H-TaS_2_ and metallic H-TaSe_2_. Rectangles are used to indicate the data range that has been extrapolated from the literature for Δ*G*_H_ (refs (25), (39), (41), (42), and (45)).

In this context, the transition-metal dichalcogenides (TMDs), made of covalently bonded C–M–C units (M = transition metal; C = chalcogen, i.e., S, Se, Te),^19,20^ have attracted strong interest for the HER.^21−24^ Theoretical^25−27^ and experimental^28−31^ investigations have shown that the HER active sites of the natural semiconducting phase (2H) of molybdenum (Mo)- and tungsten (W)-based TMDs are chalcogen-unsaturated edges, since they have a close to zero Gibbs free energy for the atomic H adsorption (Δ*G*_H_) in acidic condition. To fully exploit such high per site HER activity, the controllable synthesis of nanostructured TMDs^27,29,32−34^ has been pursued to maximize the number of the catalytically active edges.^35−37^ The designed nanostructured TMDs have shown the possibility to reach overpotential at a cathodic current density of 10 mA cm^–2^ (η_10_) inferior to 0.1 V, approaching that of noble-metal-based electrocatalysts.^35−37^ However, the complex material nanostructuring unavoidably tackles cost and scalability concerns, pointing out the need of alternative strategies. Recently, the metallic 2H-TMDs based on group 5 metals (i.e., tantalum (Ta), niobium (Nb), and vanadium(V)) have raised paramount appeal for the HER because of their intrinsic basal plane activity (especially for the sulfides)^38−44^ that is beyond that of either metal or chalcogen edges.^41−44^ The latter statement has been confirmed by density functional theory (DFT) calculations, whose outcomes are summarized in Figure 1.^25,39,41,42,45^ Clearly, the catalytic properties of their basal planes could make these materials compatible with scalable existing electrode designs. To date, 2H-TaS_2_ nanoplatelets synthesized by chemical vapor deposition (CVD) have displayed record-high surface HER activity (e.g., η_10_ < 60 mV with a loading of the catalyst <60 μg cm^–2^) among all of the reported TMDs.^40^ However, such Pt-competing HER activity has been achieved after a peculiar electrochemical treatment, namely, thousands of cyclic voltammetry (CV) scans. This peculiar behavior is still under debate, and it has been mainly attributed to morphological changes of the 2H-TaS_2_ nanoplatelets.^40^ More in detail, theoretical/experimental results on CVD-synthesized 2H-TaS_2_ nanoplatelets supported a cycling-induced self-optimizing morphology evolution from thick to thin platelets without any noticeable changes neither in the crystal structure nor in the chemical composition of the materials.^40^ Such morphological changes have been associated with a fastening of electron transport with shortening of the interlayer electron-transfer pathways in thin samples, as well as to an improved accessibility of aqueous proton (H_3_O^+^) to the catalytic sites.^40^ However, such self-optimizing fragmentation could cause degradation of the electrode in absence of polymeric binding agents (e.g., perfluorosulfonic acid, Nafion).^46^ In particular, the catalyst fracturing could affect the adhesion of the catalytic film to the electrode, and the maximum HER activity could be progressively degraded after reaching the optimal electrode morphology.^46^ Furthermore, other works claimed that the surface oxides formed on the air-exposed TaS_2_ surface are peeled off by H_2_ bubbles as the HER proceeds. Therefore, the real HER activity of the 2H-TaS_2_ is exhibited subsequently.^41^ Though, clear experimental evidence of this effect and the absence of surface oxidation are still lacking, especially for electrodes using mass loading relevant for real electrolyzers. Accordingly, further understanding of the processing and use of the metallic group 5 TMDs for the HER are required for their practical prototype validation.

In this work, we produce single/few-layer H-TaS_2_ flakes (i.e., 1H-TaS_2_ monolayers and 2H-TaS_2_ flakes) by an eco-friendly liquid-phase exfoliation (LPE) of the material crystals synthesized through direct synthesis. To activate H-TaS_2_ flakes for the HER, the morphology of H-TaS_2_ films is texturized by a thermal treatment in a H_2_-rich atmosphere. Our method is compared to the electrochemical treatment, namely, 1000 CV cycles, reported in the literature^40,41^ and patent,^47^ to design an efficient catalyst for the HER based on TMDs. Lastly, accordingly to ab initio molecular dynamic (AIMD) simulations and DFT calculations,^42^ the H-TaS_2_ flakes are hybridized with the H-TaSe_2_ flakes (produced similarly to H-TaS_2_ flakes) to tune the Δ*G*_H_ of the H-TaS_2_ basal planes and edges in the resulting heterogeneous configurations toward the ideal close to zero value in acidic media. In agreement with the theoretical expectations, in acidic media (0.5 M H_2_SO_4_), the proposed heterogeneous catalysts, based only on group 5 TMDs, outperform their single counterparts. In addition, the heterogeneous catalysts exhibit a high mass activity of 314 A g^–1^ at an overpotential of 200 mV, which is promising for practical applications. For the first time, the HER activity of these materials is investigated in alkaline media (1 M KOH), showing a η_10_ of 230 mV and a mass activity of 220 A g^–1^ at an overpotential of 300 mV (for the heterogeneous catalysts). The HER activity of the heterogeneous electrodes is demonstrated over several hours (12 h) of continuous operation at fixed potential corresponding to a starting current density of −80 mA cm^–2^, proving their durability. Additional characterization after electrochemical tests provides new understanding on chemical modifications during operation of these catalysts for the HER. Our results furnish a novel guidance to use the metallic group 5 TMDs as efficient HER catalysts by means of scalable material preparation and electrode processing.

## 2 Experimental Section

### 2.1 Materials

Tantalum (99.9%, <100 μm), sulfur (99.999%, <6 mm), and selenium (99.999%, 2–4 mm) were purchased from Strem, USA. Sulfuric acid (99.999%), KOH (reagent grade, 90%, flakes), Pt/C (20 wt % loading), and Nafion solution (5 wt %) were supplied by Sigma-Aldrich. The single-walled carbon nanotubes (SWCNTs) (>90% purity) were supplied by Cheap Tubes.

### 2.2 Synthesis and Exfoliation of the Crystals

The 2H-TaS_2_ and the 2H-TaSe_2_ crystals were produced by direct synthesis from their composing elements. A quantity of Ta (10 g) and chalcogen powders with a Ta:S or Ta:Se stoichiometry of 1:2 was placed in a quartz glass container (20 mm × 120 mm). After reaching high vacuum (1 × 10^–3^ Pa), the container was heated to 450 °C for 12 h and then to 600 °C for 48 h. Lastly, the Ta dichalcogenides were treated at 900 °C for 48 h and cooled down at room temperature over 24 h. The H-TaS_2_ and the H-TaSe_2_ flakes were obtained through LPE,^48,49^ followed by sedimentation-based separation (SBS), in 2-propanol (IPA) of the as-synthesized crystals. More in detail, 50 mg of fragmentized crystals was inserted in 50 mL of anhydrous IPA. The so-obtained mixture was ultrasonicated in a sonicator (Branson 5800 cleaner, Branson Ultrasonics) for 6 h. Afterward, the dispersion was ultracentrifuged using a Beckman Coulter centrifuge (Optima XE-90 with a SW32Ti rotor) at 2700*g* for 20 min at 15 °C in order to separate the exfoliated materials in the supernatant from the unexfoliated bulk crystals, which was found as sediment. Finally, the exfoliated materials were collected by pipetting 80% of the supernatant, thus getting the exfoliated Ta dichalcogenide dispersion. The concentration of H-TaS_2_ and H-TaSe_2_ flake dispersions were 0.35 and 0.3 g L^–1^, respectively.

### 2.3 Preparation of the Dispersions of the Exfoliated Materials

The dispersions of H-TaS_2_ and H-TaSe_2_ flakes were used as produced. The hybrid dispersions of H-TaS_2_ and H-TaSe_2_ flakes were produced by mixing the H-TaS_2_ flakes and H-TaSe_2_ flakes dispersions (material weight ratio of 1:1). The dispersion of SWCNTs in *N*-methyl-2-pyrrolidone (NMP) was produced following the protocols based on ultrasonication-based debundling,^50,51^ as previously reported in the literature.^24,36,37,42^ To produce the dispersion of Pt/C, 5 mg of Pt/C was dissolved in 950 μL of ethanol and 50 μL Nafion solution. The Pt/C dispersion was ultrasonicated for 5 min before being used.

### 2.4 Characterization of the Materials

Scanning electron microscopy (SEM) analysis of the as-synthesized crystal and the exfoliated materials was performed using a Helios Nanolab 600 DualBeam microscope (FEI Company) and 10 kV and 0.2 nA as measurement conditions. The energy-dispersive X-ray spectroscopy (EDS) spectra were acquired with a microscope combined with an X-Max detector and INCA system (Oxford Instruments) operating at 15 kV and 0.8 nA. The samples were imaged without any metal coating or pretreatment. Transmission electron microscopy (TEM) images were acquired with a JEM 1011 (JEOL) TEM (thermionic W filament) operating at 100 kV. ImageJ software (NIH) and OriginPro 9.1 software (OriginLab) were used to perform the morphological and statistical analyses, respectively. The samples were produced by depositing the exfoliated material dispersions onto ultrathin C-on-holey C-coated Cu grids. The grids were then rinsed with deionized water and subsequently dried overnight under vacuum. Atomic force microscopy (AFM) measurements were carried out using Nanowizard III (JPK Instruments, Germany) mounted on an Axio Observer D1 (Carl Zeiss, Germany) inverted optical microscope. The measurements were carried out using PPP-NCHR cantilevers (Nanosensors, USA) having a tip with a nominal diameter of 10 nm. A drive frequency of ∼295 kHz was used for image acquisition. The images were collected in intermittent contact mode over an area of 2.5 × 2.5 μm^2^ (512 × 512 data points) using a scan rate of 0.7 Hz. The working set point was set above 70% of the free oscillation amplitude. The height profile analysis was performed using the JPK Data Processing software (JPK Instruments, Germany). OriginPro 9.1 software was used to perform the statistical analysis of the thickness of the flakes, which were visualized on multiple AFM images acquired for each sample. The samples were produced by depositing the exfoliated material dispersions on mica substrates (G250-1, Agar Scientific Ltd.). Before the measurements the samples were dried under vacuum overnight. PANalytical Empyrean using Cu Kα radiation was used to perform X-ray diffraction (XRD) measurements. The samples were produced by depositing the exfoliated material dispersions onto substrates of Si/SiO_2_. Before the measurements, the samples were dried under vacuum overnight. Renishaw microRaman Invia 1000, mounting a 50× objective and using an excitation wavelength of 532 nm and an incident power on the samples of 1 mW, was used to carry out the Raman spectroscopy measurements. The samples were prepared by depositing the exfoliated material dispersions onto substrates of Si/SiO_2_. Before the measurements the samples were dried under vacuum overnight.

### 2.5 Fabrication of the Electrodes

The electrodes were fabricated by sequentially depositing the SWCNTs and exfoliated catalytic material (H-TaS_2_, H-TaSe_2_, and H-TaS_2_:H-TaSe_2_) dispersions onto commercial Whatman membrane filters (nylon with a pore size of 0.2 μm) through the vacuum filtration method (electrode area = 3.8 cm^2^). The material mass loadings were ∼1.31 and ∼0.20 mg cm^–2^ for the SWCNTs and the exfoliated catalytic materials, respectively. Before the electrochemical measurements, the electrodes were dried at room temperature overnight. The electrodes were thermally treated in a quartz tube (inner diameter = 25 mm, length = 120 cm) placed in a 3-zone split furnace (PSC 12/–/600H, Lenton, UK). While keeping a 100 sccm flow of Ar(90):H_2_(10) gas mixture through the tube, the electrodes were heated at 600 °C with a ramp of 12 °C min^–1^ for 3 h. An array of mass flow controllers (1479A, mks, USA) was used to control upstream the flow of the gases. Lastly, the furnace was switched off, and the quartz tube was cooled down to room temperature. Electrodes made entirely of SWCNTs were also produced as reference. The electrodes of Pt/C were fabricated by drop casting the Pt/C dispersion onto cleaned glassy carbon (GC) sheets. The mass loading of Pt/C was 0.354 mg cm^–2^.

### 2.6 Characterization of the Electrodes

The SEM imaging of the as-produced electrodes and the electrodes after CV cycling was performed using the microscope and the parameters reported for material characterization. The SEM-coupled EDS analysis of the electrodes was performed using a field-emission scanning electron microscope (JEOL JSM-6490LA SEM). The acceleration voltage was set to 25 kV. X-ray diffraction measurements were acquired with a PANalytical Empyrean using Cu Kα radiation. The electrochemical measurements of the electrodes were performed using a VMP3 multichannel potentiostat/galvanostat (Bio-Logic) controlled via Bio-Logic’s own software. The measurements were carried out in a three-electrode configuration at room temperature and using a footed 250 mL quartz cell with dual flat windows (Pine Research) as the electrochemical cell. A KCl-saturated Ag/AgCl and a carbon rod and were used as the reference electrode and the counter electrode, respectively. The measurements were performed in acid (0.5 M H_2_SO_4_) or alkaline (1 M KOH) media (medium volume = 200 mL). Inductively coupled plasma optical emission spectroscopy (ICP-OES) measurements were carried out to evaluate the contamination in the KOH reagent. These measurements were carried out using an iCAP 6000 Duo (Thermo Fisher Scientific) on a sample prepared by digesting 25 mg of KOH in 2.5 mL of HCl:HNO_3_ (3:1 vol/vol) overnight. Before starting the electrochemical measurements, N_2_ gas was flowed throughout the liquid media using a porous frit in order to remove the dissolved O_2_. The applied/measured potentials vs Ag/AgCl were converted to the reversible hydrogen electrode (RHE) scale according to the Nernst equation *E*_RHE_ = *E*_Ag/AgCl_ + 0.059 × pH + *E*^0^_Ag/AgCl_ in which *E*_RHE_ is the potential vs the RHE, *E*_Ag/AgCl_ is the potential vs the Ag/AgCl reference electrode, and *E*^0^_Ag/AgCl_ is the standard potential of the Ag/AgCl reference electrode at 25 °C (0.1976 V vs. RHE). The LSV curves were measured using a potential scan rate of 5 mV s^–1^. The LSV data were *iR* corrected (100% *iR*-drop compensation) by considering *i* as the measured working electrode current and *R* as the series resistance of the resistance of the electrolyte and the resistance of the substrate of the working electrode. *R* was measured through electrochemical impedance spectroscopy (EIS) measurements at open-circuit potential and using a frequency of 10 kHz. The mass activity of the catalytic films was evaluated by the ratio between the current density measured at fixed potential and the catalyst mass loading. The mass loading of the catalysts was approximated to that of the electrode before any kind of treatments. Chronoamperometry measurements were carried to evaluate the stability of the electrodes. The overpotential was set to provide an initial cathodic current density of 80 mA cm^–2^. An alkaline-resistant flat-bottom polytetrafluoroethylene (PTFE) (Pine Research) cell was used for the stability tests in alkaline media in order to exclude quartz dissolution effects on the electrode performance.

## 3 Results and Discussion

### 3.1 Production and Characterization of H-TaS_2_ Flakes

**Figure 2:**
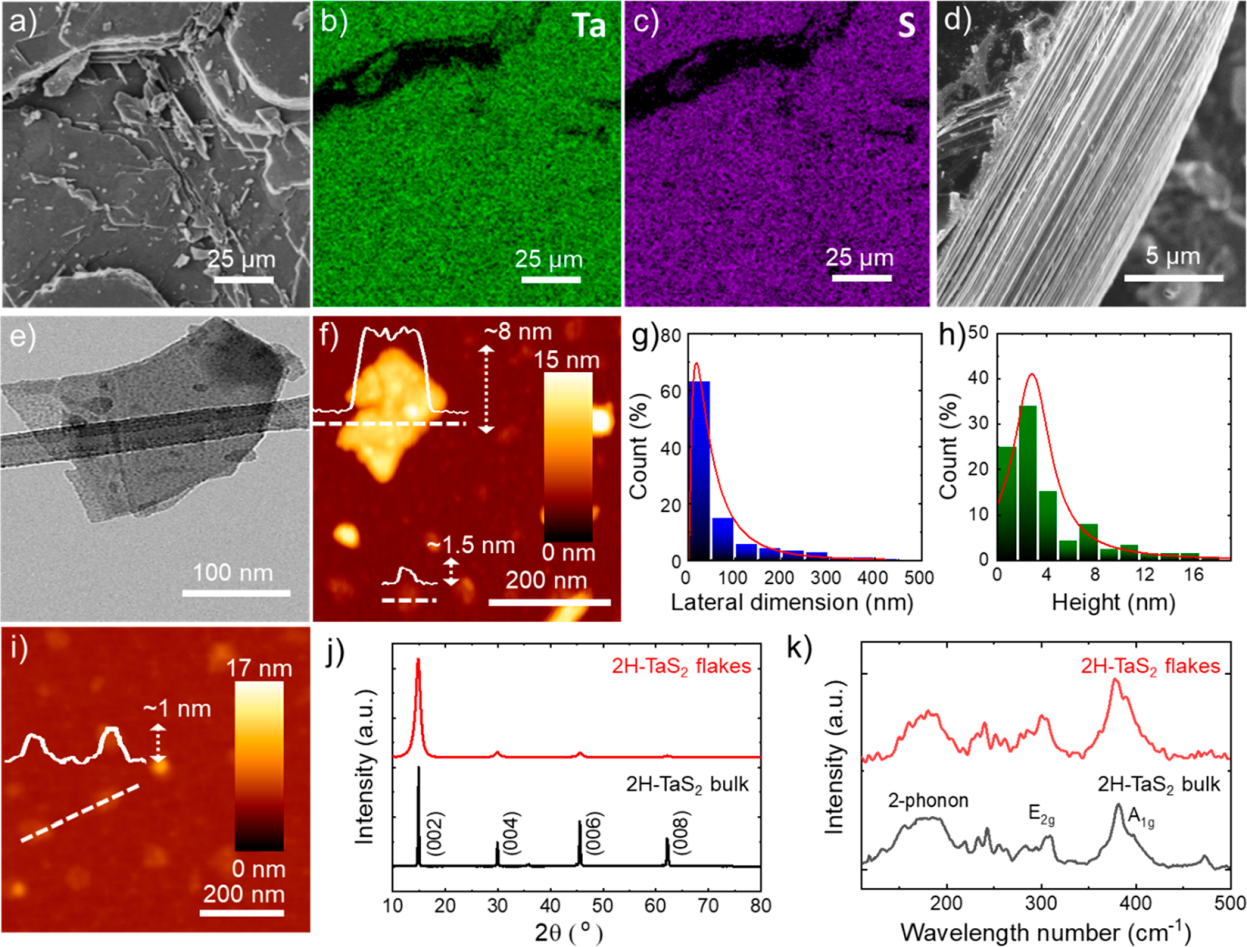
(a) SEM image of as-synthesized 2H-TaS_2_ crystals with corresponding EDS maps for (b) Ta (Lα = 8.14 keV) and (c) S (Kα = 2.3 keV). (d) High-magnification SEM image of an edge of a representative 2H-TaS_2_ crystal, evidencing its layered structure. (e) TEM image of representative H-TaS_2_ flakes. (f) Representative AFM image of representative H-TaS_2_ flakes. (g) Statistical analysis of the lateral dimension of H-TaS_2_ flakes. (h) Statistical analysis of H-TaS_2_ flakes. (i) AFM image of H-TaS_2_ flakes, showing the presence of monolayer 1H-TaS_2_ flakes. (j) XRD and (k) Raman spectra of the as-produced 2H-TaS_2_ bulk crystals and H-TaS_2_ flakes.

The 2H-TaS_2_ crystals were synthesized by the direct reaction from elements using Ta powder and S granules in a quartz glass ampule (see Experimental Section for the details). After reaction, the products were cooled down slowly in order to stabilize the 2H phase. Scanning electron microscopy-coupled EDS measurements of the as-synthesized 2H-TaS_2_ crystals (Figure 2a–c) indicate a near-ideal stoichiometric phase of the 2H-TaS_2_ crystals (S-to-Ta atomic % ratio = 1.9, see Supporting Information, Table S1), as expected from previous studies.^45,52^ The high-magnification SEM image (Figure 2d) of the edges of a crystal clearly evidence the layered structure expected for 2H-TaS_2_.

The H-TaS_2_ flakes were produced by LPE^48,49^ of the synthesized crystals in IPA followed by SBS^53,54^ to remove the unexfoliated material (see Experimental Section for additional details). Our approach aimed to provide a scalable method to produce nanostructured H-TaS_2_ starting from cost-effective synthesized crystals and without resorting time-consuming bottom-up nanomaterial synthesis, such as CVD, or complex processing of materials.^55^

The morphology of H-TaS_2_ flakes was characterized by TEM and AFM. Figure 2e reports the TEM image of representative H-TaS_2_ flakes, which show irregularly shaped wrinkled structures. An AFM image of the H-TaS_2_ flakes is shown in Figure 2f, together with the height profiles of two individual 2H-TaS_2_ flakes with thicknesses of ∼1.5 and ∼8 nm, respectively. Statistical TEM analysis of the lateral dimension of the flakes (Figure 2g) shows values in the range of 10–450 nm, mainly distributed at values < 100 nm (log-normal distribution peaks at ∼30 nm). The statistical AFM analysis of the thickness of the flakes (Figure 2h) indicates that the sample is mainly made of few-layer 2H-TaS_2_ flakes (AFM thickness of a TaS_2_ monolayer is typically between 0.4 and 0.9 nm).^56−58^ Their thickness follows a log-normal distribution peak at ∼2.8 nm. The 1H-TaS_2_ monolayers have also been observed in the exfoliated sample, as reported in the AFM image shown in Figure 2i.

The metallic H phase of the TaS_2_ flakes was confirmed by XRD measurements (Figure 2j) as indexed by ICSD-651082.^52,59^ For the H-TaS_2_ flakes, the (002) peak is broader (full width half-maximum (fwhm) = 1.06°) than that of the synthesized 2H-TaS_2_ crystal (fwhm = 0.18°). The broadening of the XRD peak could be related to the extent of the crystalline domain (the broader the peak, the smaller the crystalline domain). Therefore, it indicates the successful exfoliation of the sample. The other reflections are strongly reduced in intensity, although they retain their native position. This means that the H-TaS_2_ flakes preserve their native crystal structure while orienting with their *c* axis perpendicular to the substrate.^34,60^ Raman spectroscopy measurements (Figure 2k) further confirm the crystallinity retention of the exfoliated sample, which exhibits the same Raman modes of the native crystal (e.g., the out-of-plane vibration mode A_1g_ at ∼380 cm^–1^, the in-plane vibrational mode E^1^_2g_ at ∼300 cm^–1^, and the broad second-order peak attributed to a two-phonon process at ∼180 cm^–1^).^61,62^

### 3.2 H-TaS_2_ Electrode Fabrication and Characterization

To take advantage of the production of the H-TaS_2_ flakes through LPE in dispersion form, the electrodes were obtained by sequential vacuum filtration of SWCNT and H-TaS_2_ flake (material mass loading of ∼1.31 and ∼0.20 mg cm^–2^ for SWCNTs and H-TaS_2_ flakes, respectively) through nylon filters (then used as electrode support). The production of the SWCNT dispersion and the protocol used to fabricate the electrodes are in agreement with our previous studies on TMDs-based catalysts (see Experimental Section for further description).^34−37,63^ Noteworthy, our electrode manufacturing approach is particularly effective for one/two-dimensional materials since it does not lead to any material losses (different from the case of noble-metal nanoparticles typically used as catalysts).^36,37^ Moreover, the choice of a SWCNT film (i.e., buckypaper) as the substrate relies on our previous findings showing that the porosity of such substrate promotes the adhesion of a TMD flake film without the need of ion-conducting catalyst binders.^24,36,37,64^

**Figure 3:**
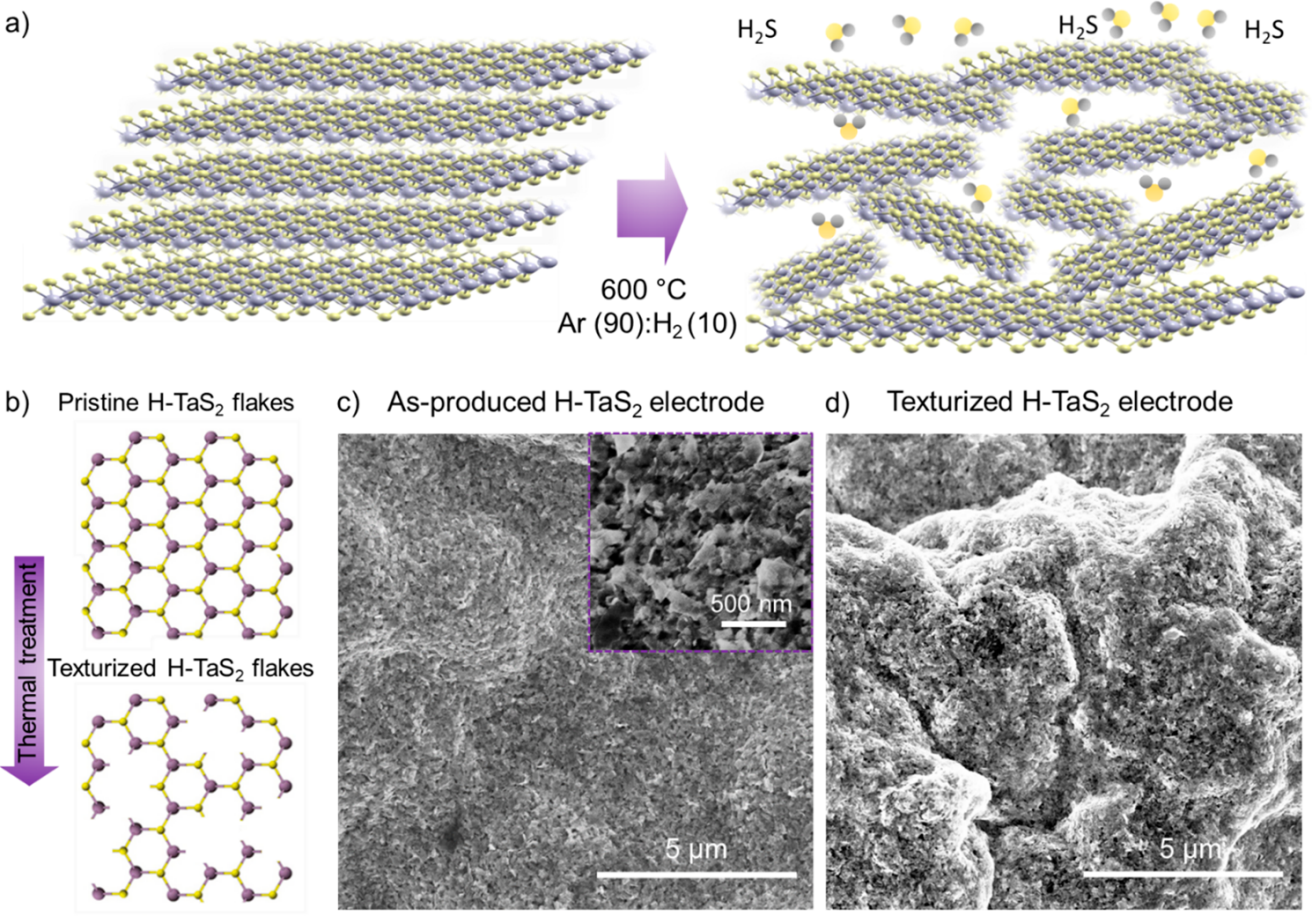
(a) Schematic illustration of the H_2_S-aided texturization of the H-TaS_2_ electrodes treated at 600 °C in H_2_-rich environment (Ar(90):H_2_(10) atmosphere). During this process, molecular H_2_ reacts with tH-TaS_2_ flakes and the S atoms are removed as H_2_S (in form of gas). Evolved H_2_S gas perforates and peels away the H-TaS_2_ flakes, causing a laminar-to-porous conversion of the electrode structure. (b) Sketch of the texturization at “flake level” in which the evolution of H_2_S gas enriches the number of highly HER active Ta edges. (c and d) Top-view SEM images of the H-TaS_2_ electrode before and after the thermal treatment at 600 °C in Ar(90):H_2_(10) atmosphere. (Inset to c) Enlargement of the surface of the as-produced H-TaS_2_ electrodes, evidencing the flake-composed laminar structure.

In order to resemble the self-optimizing texturization of the H-TaS_2_ films previously reported by electrochemical treatments (i.e., CV cycling),^40,41,44^ where H_2_ evolving from the TMD basal planes causes catalyst fracturing, our electrodes have been thermally treated in a H_2_-rich environment at 600 °C (Figure 3a) (the resulting sample is herein named H-TaS_2_-Ar/H_2_@600°C). In fact, during this process, the molecular H_2_ reacts with the H-TaS_2_ flakes and S atoms are removed as H_2_S (in the form of gas) (Figure 3b).^33^ Beyond the formation of HER active Ta edges, the H_2_S gas evolving from the basal planes perforates or peels away H-TaS_2_ layers. Consequently, this effect increases the porosity and the electrochemically accessible surface area of the electrode films.^64,65^ Double-layer capacitance (*C*_dl_) measurements of the H-TaS_2_ films (deposited on flat GC substrates in order to exclude the capacitive contribution of SWCTNs) before and after the thermal treatment were performed to confirm the effect attributed to the thermal treatments (Figure S1). These data show that the thermal treatments significantly increase (by +39%) the *C*_dl_ of the electrodes, which means that their electrochemically accessible surface area also increases. Moreover, EDS measurements of the H-TaS_2_ electrodes show an ∼14% reduction of the S content after the thermal treatment, corroborating the S removal from the H-TaS_2_ flakes via the H_2_S evolution process. The top-view SEM images of the electrode before and after the thermal treatment (Figure 3c and 3d) also confirm the above-discussed morphology evolution of the H-TaS_2_ film. By doing so, the H_2_S evolution-aided texturization partially resembles the H_2_ evolution-aided one performed by the in-operando electrochemical approach.^40,41^ However, our method does not require time-consuming electrochemical conditioning and simultaneously creates highly HER active metallic edges. In addition to the morphology changes, XRD measurements reveal the formation of oxides (i.e., Ta_2_O_5_) onto the surface of thermally treated H-TaS_2_ films (Figure S2). In agreement with previous studies on other TMDs (namely, 2H-MoS_2_),^65^ the chalcogen loss can lead to the formation of elemental metal, which subsequently oxidizes when it is exposed to air. Moreover, surface-sensitive grazing angle XRD measurements have shown that this process mainly affects the surface of the material in contact with H_2_-rich atmosphere, while the remaining material can preserve its chemical properties,^65^ allowing the flakes to not drastically evolve from a two-dimensional morphology to cluster-like structures (caused by metal coalescence), in agreement with our SEM analysis (Figure 3).

**Figure 4:**
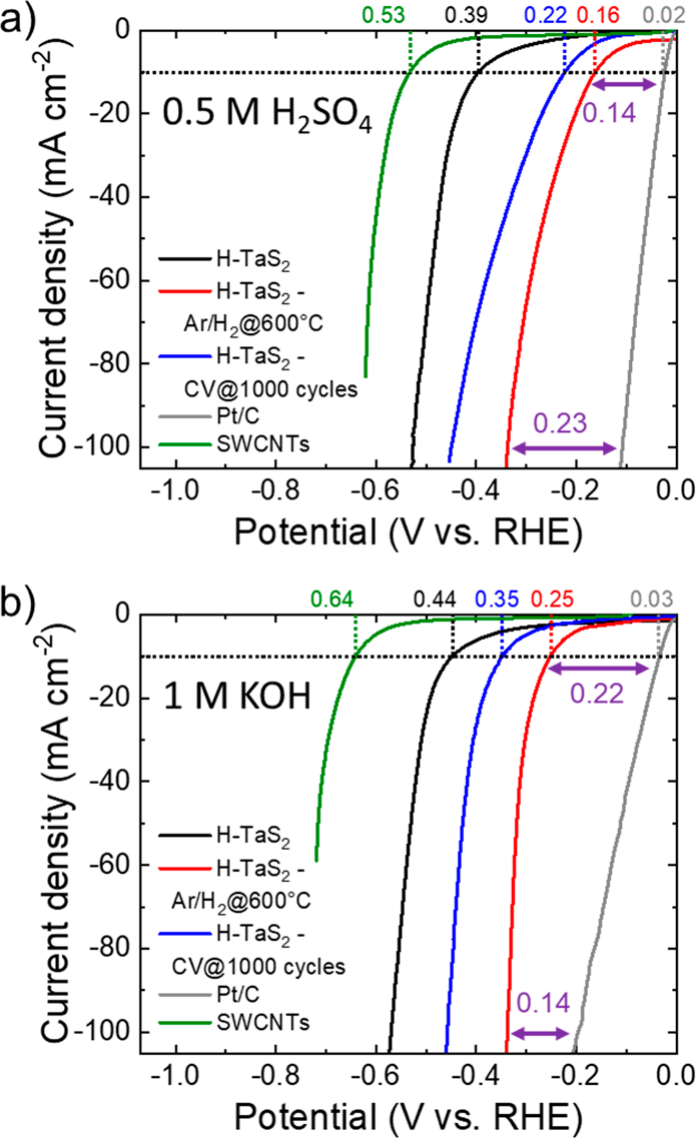
(a and b) *iR*-corrected LSV curves measured for H-TaS_2_, H-TaS_2_-Ar/H_2_@600°C, and H-TaS_2_-CV@1000 cycles in 0.5 M H_2_SO_4_ and 1 M KOH, respectively. LSV curves measured for the SWCNTs (catalyst support) and the Pt/C (benchmark) are also plotted for comparison. η_10_ values measured for the electrodes are indicated. For H-TaS_2_-Ar/H_2_@600°C, the HER overpotentials vs the overpotential of Pt/C at 10 and 100 mA cm^2^ are also shown.

The HER activity of the H-TaS_2_ electrodes was investigated in either acidic (0.5 M H_2_SO_4_) or alkaline (1 M KOH) N_2_-purged solutions at a temperature of 22 °C (room temperature). To the best of our knowledge, the HER activity of the Ta dichalcogenides in alkaline conditions was not studied neither theoretically nor experimentally. Figure 4a and 4b shows the *iR*-corrected linear sweep voltammetry (LSV) curves in 0.5 M H_2_SO_4_ and 1 M KOH, respectively, for the investigated electrodes before and after the thermal treatment (samples named H-TaS_2_ and H-TaS_2_-Ar/H_2_@600°C, respectively). Moreover, the LSV curves measured for the electrochemically treated electrode (i.e., nonthermally treated electrode after 1000 CV cycles, sample named H-TaS_2_-CV@1000 cycles), the SWCNTs (catalyst support), and the Pt/C (benchmark) are also shown. In 0.5 M H_2_SO_4_, H-TaS_2_-Ar/H_2_@600°C exhibits a HER activity significantly higher than that of the as-produced electrodes (H-TaS_2_). In particular, H-TaS_2_-Ar/H_2_@600°C shows a η_10_ of 160 mV, which is also inferior to that of the H-TaS_2_-CV@1000 cycles (η_10_ = 220 mV). Similar results were measured in 1 M KOH, in which H-TaS_2_-Ar/H_2_@600°C shows a η_10_ of 250 mV, whereas the as-produced H-TaS_2_ and H-TaS_2_-CV@1000 cycles display a η_10_ of 440 and 350 mV, respectively. A thorough analysis of the HER kinetics, including the extrapolation of both the Tafel slope and the exchange current, was not carried out in this work because misleading interpretations can derive from the presence of the highly porous SWCNTs as the support of our catalytic films. In fact, SWCNTs have a high electrochemically accessible surface area that causes a significant capacitive current density (in the order of 1 or 10 mA cm^–2^) even at low potential scan rate (i.e., <10 mV s^–1^).^36^ Such capacitive contribution of the substrate is often the cause of mistakes, since it makes the correct evaluation of the kinetic parameters through standard protocols hard (see further details above in the text, section 3.4, which will specifically discuss the intrinsic catalytic properties of our electrodes).^66,67^ Noteworthy, the differences between the HER overpotential of the Pt/C and the H-TaS_2_-Ar/H_2_@600°C electrode at a current density of 100 mA cm^–2^ is as low as 230 and 0.140 mV in 0.5 MH_2_SO_4_ and 1 M KOH, respectively. In agreement with previous studies on group 5 TMDs,^68^ these results indicate that our electrodes may optimally operate at high current densities, such as those required in real electrolyzers. Moreover, our results agree with the recent DFT simulations,^25,45^ which show that the atomic H binding for both Ta and S edges (displaying Δ*G*_H_ < 0 eV at low atomic H coverage, i.e., ≤25%, see also Figure 1) weakens incrementally with increasing H coverage, leading to ideal-like Δ*G*_H_ close to 0 eV. Overall, our data suggests that H-TaS_2_ flakes may be efficient and scalable HER catalysts.

### 3.3 H-TaS_2_:H-TaSe_2_ Heterogeneous Catalysts

**Figure 5:**
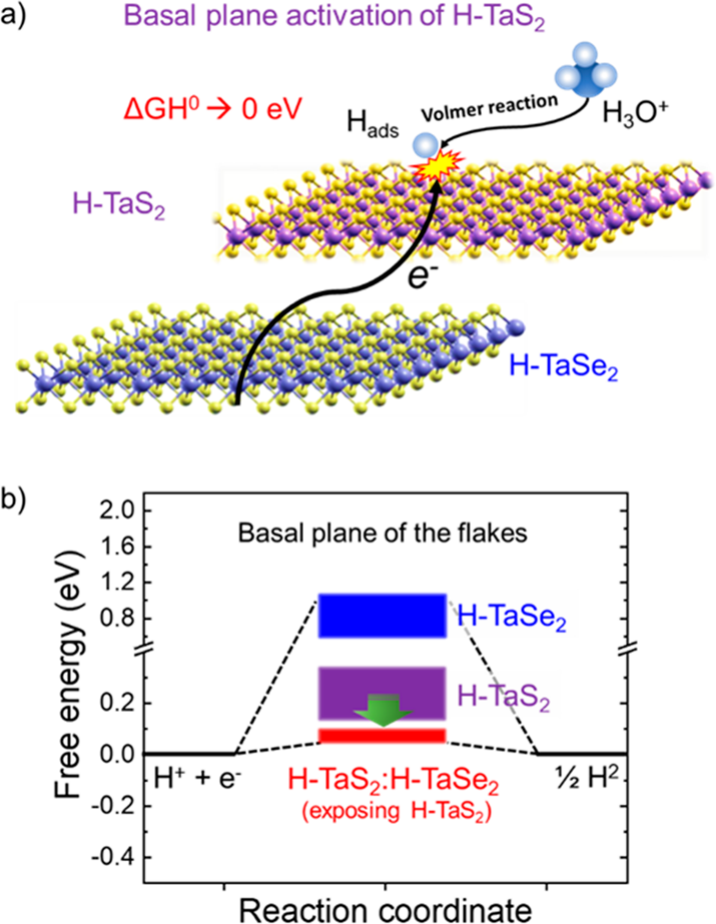
(a) Schematic illustration of the activation of the H-TaS_2_ flakes for the HER in 0.5 M H_2_SO_4_ via the hybridization of H-TaS_2_ and H-TaSe_2_ flakes. Hybridization process fosters an electron transfer from H-TaSe_2_ flakes toward H-TaSe_2_ flakes, decreasing the standard Δ*G*_H_ value of the basal plane of H-TaS_2_ flakes toward 0 eV. This effect promotes the first step of the HER (Volmer reaction, i.e., H_3_O^+^ + e^–^ ⇄ H_ads_ + H_2_O). (b) Evolution of the standard Δ*G*_H_ for the basal planes of H-TaS_2_, H-TaSe_2_ flakes, and H-TaS_2_:H-TaSe_2_ heterogeneous catalysts. Rectangles are used to indicate data ranges that have been extrapolated from the literature for the standard Δ*G*_H_ (ref (42)).

To further utilize the potential of group 5 TMDs for the HER, H-TaS_2_ flakes were hybridized with the TaSe_2_ flakes to tune the Δ*G*_H_ of resulting heterogeneous configurations to optimal close to zero values for both the edges and the basal planes of the flakes. Although the theoretical HER activity of Se-based group 5 TMD has been shown to be lower than that of S-based counterparts,^40^ recent DFT simulations and AIMD simulations^42^ revealed that the heterogeneous stacking of Se- and S-based group 5-TMDs can increase the HER activity of S-based parts. Particularly for the case of Ta-based TMDs, the stacking promotes an electron transfer from H-TaSe_2_ flakes to H-TaS_2_ flakes (Figure 5a), decreasing the standard Δ*G*_H_ of the H-TaS_2_ basal plane (>0.1 eV) toward 0 eV (Figure 5b).^42^ Although the standard Δ*G*_H_ of the basal planes of stacked H-TaS_2_ flakes might still be higher than 0 eV (and higher than the nearly zero standard Δ*G*_H_ of the edge sites), the abundance of the HER active sites associated with their basal planes could promote the HER activity at high H coverage conditions (i.e., high current density). This effect could make such heterogeneous catalysts competitive with metallic catalyst benchmarks, including Pt/C.^42^ In addition, the hybridization approach can preserve the scalability of the catalysts preparation, since it does not require in any complex morphological/structural chemical modifications, such as the chemical doping of heteroatoms, the creation of artificial defects, or the strain impositions.^42^

The details regarding the synthesis of the 2H-TaSe_2_ crystals are reported in the Experimental Section. Figure S3a–c shows the SEM-coupled EDS analysis of the as-synthesized 2H-TaSe_2_ crystal, revealing a near-ideal stoichiometric phase (Se to Ta atomic % ratio = 2.2, see Table S2), which agrees with the previous literature.^45,52^ The layered structure of the 2H-TaSe_2_ crystals is evidenced on its edges, as proven by a representative high-magnification SEM image (Figure S2d). The H-TaSe_2_ flakes were produced through LPE of fragmentized 2H-TaSe_2_ crystals in IPA, following the same protocol used for the exfoliation of 2H-TaS_2_ crystals. Transmission electron microscopy (Figure S4) and SEM-coupled EDS (Figure S5) analyses show that the exfoliated sample consists of H-TaSe_2_ flakes and one-dimensional trigonal Se byproducts. The latter are formed by the dissolution–recrystallization mechanism involving polycrystalline Se,^69−71^ whose excess has been also detected in the as-synthesized crystals (see Figure S3).

Following the protocols used for H-TaS_2_ electrodes, H-TaSe_2_ and heterogeneous H-TaSe_2_:H-TaS_2_ (material mass ratio of 1:1) electrodes (hereafter named H-TaSe_2_ and H-TaSe_2_:H-TaS_2_) were fabricated through vacuum filtration of their dispersions onto SWCNTs. Figure S6 reports representative SEM images of the H-TaSe_2_ and H-TaSe_2_:H-TaS_2_ electrodes. The as-produced electrodes display a wrinkled structure, which is different from the laminar one shown for the H-TaS_2_ electrodes. In fact, the whiskers in the exfoliated H-TaSe_2_ sample modify the arrangement of the flakes during their film deposition. Although it is realistic to suppose that the vacuum filtration deposition of a dispersion of a mixture of H-TaS_2_ and H-TaSe_2_ nanoflakes naturally leads to the formation of some stacks between nanoflakes of different materials, SEM-coupled EDS measurements were performed to demonstrate the absence of single-material domains. Top-view and cross-sectional SEM-coupled EDS analyses of the heterogeneous films composed by H-TaS_2_ and H-TaSe_2_ flakes (Figure S7) show homogeneous distributions for both S and Se, which indicates an optimum material mixing. Although our approach cannot accurately control the formation of heterogeneous configurations alternating flakes of different materials, we point out that it is promptly scalable and time saving compared to highly controlled nanofabrication methods. Moreover, for practical mass loadings, such as those used for our electrodes (i.e., 0.2 mg cm^–2^), a highly controlled stacking of flakes of different materials is problematic with any method. Therefore, our method is convenient to design an efficient TMD-based electrode with a high mass loading of the catalysts. A similar approach has been recently used to make heterogeneous stacking between H-NbS_2_ and H-MoSe_2_ flakes to design heterogeneous catalysts with HER performance superior to those of the single catalytic counterpart.^43^

The thermal treatment of H-TaSe_2_-based electrodes in a H_2_-rich environment at 600 °C causes the evolution of H_2_Se (in form of gas), in agreement with previous studies on another Se-based TMD (i.e., H-MoSe_2_).^64^ Therefore, the effects of the thermal treatment on H-TaSe_2_ electrodes resemble those occurring on H-TaS_2_ electrodes (see Figure 3). Moreover, XRD measurements on H-TaSe_2_ films reveal the presence of surface oxides after the thermal treatment (Figure S8), similarly to the case of H-TaS_2_ or other TMDs (e.g., 2H-MoS_2_).^65^ Lastly, it is worth noticing that for H-TaSe_2_ both Ta and Se edges have been theoretically predicted to be highly catalytic for the HER process, whereas the basal planes are deemed inactive (differently from the H-TaS_2_).^20,38,39,64^

**Figure 6:**
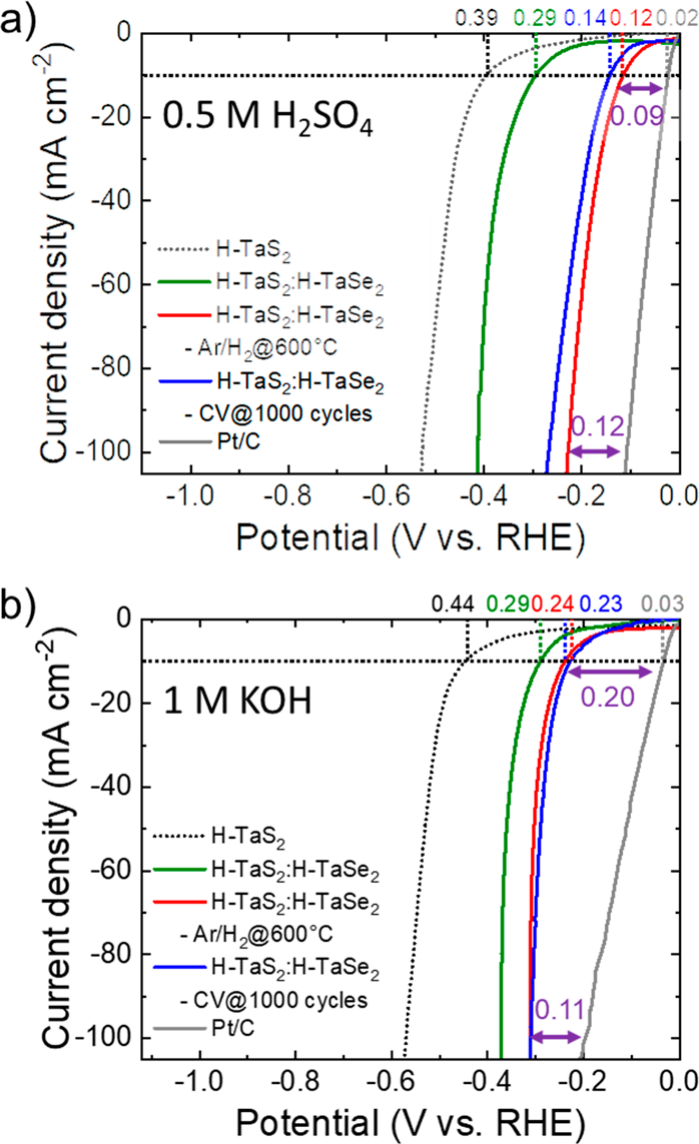
(a and b) *iR*-corrected LSV curves measured for H-TaS_2_:H-TaSe_2_, H-TaS_2_:H-TaSe_2_-Ar/H_2_@600°C, and H-TaS_2_:H-TaSe_2_-CV@1000 cycles in 0.5 M H_2_SO_4_ and 1 M KOH solutions, respectively. LSV curves measured for Pt/C and H-TaS_2_ (reference, dashed line) are also shown for comparison. η_10_ values measured for the electrodes are indicated. For H-TaS_2_:H-TaSe_2_-Ar/H_2_@600°C, the HER overpotentials vs the overpotentials of Pt/C at 10 and 100 mA cm^–2^ are also shown.

Figure 6a and 6b shows the LSV curves in 0.5 M H_2_SO_4_ and 1 M KOH, respectively, for the as-produced heterogeneous electrodes before and after the thermal treatment (samples named H-TaS_2_: H-TaSe_2_ and H-TaS_2_:H-TaSe_2_-Ar/H_2_@ 600 °C). Furthermore, the LSV curves measured for the nonthermally treated electrode after 1000 CV cycles (sample named H-TaS_2_:H-TaSe_2_-CV@1000 cycles), the H-TaS_2_ electrode (reference), and the Pt/C (benchmark) and are also plotted. Electrochemical characterization of the electrodes made of only H-TaSe_2_ flakes before and after thermal or electrochemical treatments is reported in the Supporting Information (Figure S9). As predicted by theoretical DFT simulations (see Figure 1),^20,38,39^ H-TaSe_2_ electrodes exhibit relevant HER activities in both acidic and alkaline media. In particular, after thermal treatment, the electrodes show a η_10_ as low as 200 and 260 mV in 0.5 M H_2_SO_4_ and 1 M KOH, respectively. These HER activities can be attributed to the abundant Ta edges, as detected by the EDS analysis (Figure S5). The hybridization of H-TaS_2_ and H-TaSe_2_ flakes increases the HER activity of both H-TaS_2_ and H-TaSe_2_ electrodes_._ Similarly to the single counterparts, both thermal treatment and CV cycling enhance the HER activity of our heterogeneous electrodes. In acidic condition, H-TaS_2_:H-TaSe_2_-Ar/H_2_@600°C exhibits a η_10_ of 120 mV, whereas the nonthermally treated heterogeneous electrode after 1000 CV cycles (i.e., H-TaS_2_:H-TaSe_2_-CV@1000 cycles) shows a slightly higher η_10_ (140 mV). In 1 M KOH, H-TaS_2_:H-TaSe_2_-Ar/H_2_@600°C shows a η_10_ of 240 mV, which is similar to that of H-TaS_2_:H-TaSe_2_-CV@1000 cycles (230 mV). Interestingly, at the high current density of 100 mA cm^–2^, the H-TaS_2_:H-TaSe_2_-Ar/H_2_@600°C displays low HER overpotentials, only 120 and 110 mV higher than those of the Pt/C electrode in 0.5 M H_2_SO_4_ and 1 M KOH, respectively. Although the HER activity of our heterogeneous electrodes at low current density can still be mainly ascribed to the HER active edges of both H-TaS_2_ and H-TaSe_2_ flakes (in agreement with data shown in Figure 4 and Figure S9), the remarkable HER activity at high current density can be also associated with the hybridization-induced activation of the basal planes of H-TaS_2_ flakes, as previously discussed in Figure 5.

Beyond the electrocatalytic activity, the durability of a catalyst is an essential requirement for its exploitation. Figure S10 reports the chronoamperometric measurements for our thermally treated electrodes (H-TaS_2_:H-TaSe_2_-Ar/H_2_@600°C) at a potential corresponding to a starting current density of −80 mA cm^–2^ in both acidic and alkaline media. In 0.5 M H_2_SO_4_, H-TaS_2_:H-TaSe_2_-Ar/H_2_@600°C maintained 97% of the starting current densities after 12 h, therefore proving an adequate HER activity durability. Interestingly, the durable HER activity of our electrodes has been reached without the use of any binder, such as Nafion, which could prospectively increase the mechanical strength of our electrodes as the HER proceeds. In fact, mechanical stress originated from H_2_ bubbling has been shown to cause fragmentation of the group 5 TMDs.^40,41,47^ As also shown in our experiments by treating electrodes with CV cycling, catalyst fragmentation initially improves the electrochemical performance of as-produced electrodes. However, they may also involve a significant loss of catalytic materials, which should be limited for practical targets. Differently, our thermal treatment-aided texturization does not require any electrochemical conditioning of the electrodes, and the initial porosity of thermally treated electrodes could be advantageous to limit catalyst fragmentation effects while showing optimal HER activity from the beginning of operation. As shown by SEM analysis reported in Figure S11, H-TaS_2_-Ar/H_2_@600°C, in which the detection of thermo-induced texturization is easier than the one in heterogeneous electrodes, does not show any significant difference in the electrode morphology before and after CV cycling. On the contrary, the nonthermally treated electrode after 1000 CV cycles displays a fragmented surface, which is significantly different from the initial one. Noteworthy, SEM-coupled EDS analysis of our nonthermally treated heterogeneous electrodes also evidences morphology changes after CV cycling (Figure S12a). In addition, EDS mapping (Figure S12b–d) shows a slight redistribution of elements (especially for S), indicating the possibility of chemical changes at the electrode surface during HER. As shown in Figure S13, XRD measurements of H-TaS_2_:H-TaSe_2_-CV@1000 cycles further evidence chemical changes on its surface. In particular, the intensities of the Raman peaks attributed to oxides (i.e., Ta_2_O_5_) significantly increase compared to those observed in untreated samples (whose oxidation is marginal). At this stage, we cannot exclude the dissolution of the so-formed oxides in acidic media, as speculated in previous works.^41^ The choice of proper electrochemical potential for carrying out HER could be considered to control both oxidation and dissolution effects, as shown in previous work on electrocatalysts.^72^ Overall, we can state that our results partially contradict those previously reported for H-TaS_2_ electrodes in the literature,^40,41^ where it is claimed that H-TaS_2_ preserves its chemical integrity. Therefore, additional specific studies on electrodes with catalyst mass loadings similar to those of our electrodes are still needed to definitively provide better understanding regarding possible chemical changes of this kind of catalyst in acidic media. In alkaline condition, the electrode degraded during the first 4 h; thereafter, the electrode’s current density stabilized (current density equal to 81% of the initial one after 12 h) (see Figure S10). The stabilization of the current density suggests that an equilibrium between the catalytic properties and the electrochemical stability was also reached in alkaline condition. It is worth noticing that the dissolution of the quartz of the cell in alkaline media could alter the electrolyte composition, affecting the HER activity of the electrodes.^64,65^ In order to exclude these effects, the stability tests were also carried out in a alkaline-resistant PTFE cell. As shown in Figure S14, these data confirm an initial degradation of the electrodes. Subsequently, the HER activity of the electrode progressively increases over time, suggesting an evolution toward an electrochemical equilibrium, which was also observed in the quartz cell. Elemental analysis of the KOH reagent used to prepare the 1 M KOH solution was also carried out through ICP-OES measurements to evaluate the presence of transition-metal and heavy-metal impurities, which could result in a misleading interpretation of the stability/durability of investigated electrodes.^73^ Our data indicate that the content of metals (Fe, Co, Ni, Cu, Zn, Cd, Pb) is below the detection limit of the ICP equipment, agreeing with the product specification sheet provided by the material supplier (see Experimental Section for additional technical detail), i.e., Fe < 0.0005%, Zn ≤ 0.0005%, Co ≤ 0.0005%, Cu ≤ 0.0005%, and Pb ≤ 0.001%. These values suggest that the metallic impurities are marginally affecting the HER activity of our electrodes, which are instead highly dependent by the electrode treatment proposed in this work. The chemical modification and/or dissolution of electrode materials could influence the HER activity durability of the investigated electrodes in alkaline media. Currently, we cannot rule out a possible oxidation of H-TaS_2_ during HER operation in such conditions. Since the metallic Ta can oxidize in alkaline conditions,^66,67^ oxidation processes could significantly impact the electrocatalyst metal edges, which are created during the thermal treatment of our electrodes (see Figure 3). Consequently, the initial degradation could be associated with these phenomena. Similar effects have been recently observed in other group 5 TMDs, i.e., H-NbS_2_, in which a progressive oxidation of the surface of the flakes was also shown.^43^ Moreover, it is worth pointing out that alkaline condition can promote the dissolution of oxidized Ta (e.g., Ta_2_O_5_), since it tends to form soluble oxotantalate (TaO)(OH)*_x_*^(3–*x*)+^ and hydroxotantalate (TaO)(OH)*_x_*^(5–*x*)+^.^66,68^ Consequently, a progressive reactivation of the electrode surface toward HER may explain the subsequent stabilization/increase of the HER activity of the electrodes, until the achievement of an electrochemical equilibrium. Lastly, hydr(oxy)oxide species onto HER catalyst can also synergistically interact with the latter to modify the HER activity of the electrodes. In particular, it has been recently demonstrated that transition-metal oxides (or hydroxides) on a TMD surface can increase the HER activity of the pristine TMDs in alkaline media,^37,36,74,75^ similarly to what is observed in noble metal-based electrocatalysts.^76−79^ Therefore, the control of the oxidation effects, including those occurring during our thermal treatment (see Figurea S3 and S8), could be crucial for optimizing our current electrodes. Further measurements are still needed to unambiguously demonstrate the durability of Ta-based dichalcogenides in alkaline media. However, our preliminary results open the way toward the use of Ta-based dichalcogenides in electrolyte beyond the acidic ones, as recently reported for the most established HER active transition-metal dichalcogenides, including Mo-based ones.^24,36,37,74,80^

### 3.4 Evaluation of the Intrinsic Activity of the Catalysts

**Figure 7:**
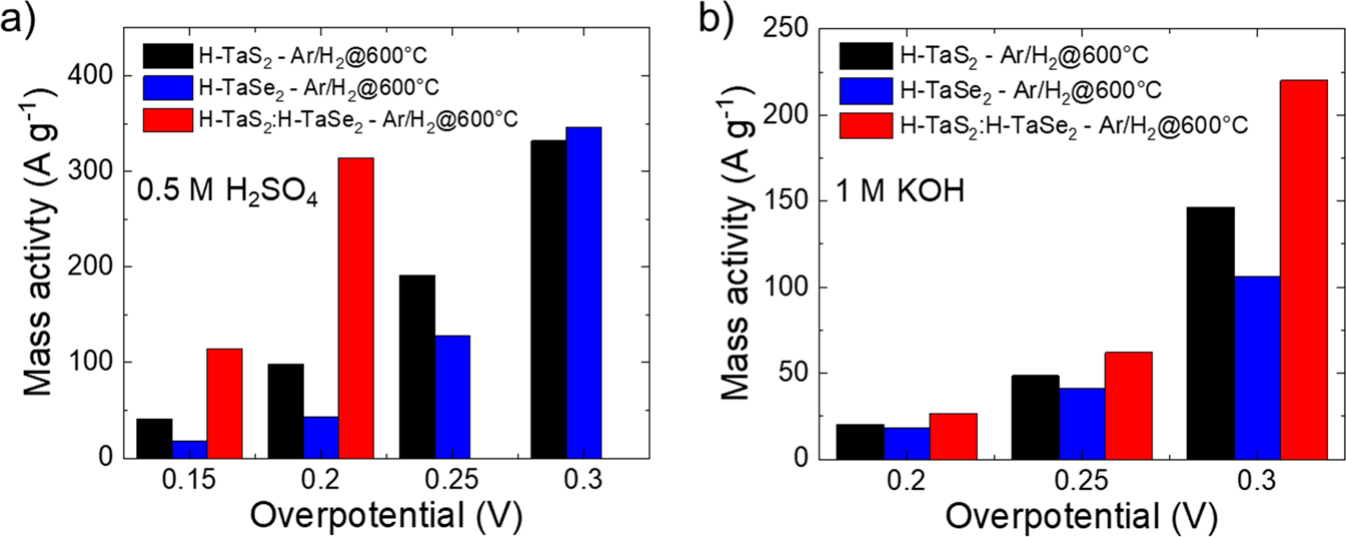
Mass activity of H-TaS_2_-Ar/H_2_@600°C, H-TaSe_2_-Ar/H_2_@600°C, and H-TaS_2_:H-TaSe_2_-Ar/H_2_@600°C catalytic films at different overpotential (vs RHE) in (a) 0.5 M H_2_SO_4_ and (b) 1 M KOH.

In order to compare the catalytic performance of our electrocatalysts with those reported in the literature for similar materials, it is fundamental to evaluate the parameters that reflect the intrinsic electrocatalytic properties.^73,81,82^ Since our catalytic films were deposited onto highly porous SWCNT films (i.e., buckypapers) as the substrates, the evaluation of the electrochemical surface area (hereafter denoted ECSA) of our electrocatalysts through traditional methods used for TMD-based electrocatalysts (e.g., C_dl_ estimation through CV measurements at different scan rate in a nonfaradaic region)^83^ can lead to overestimated values. Consequently, intrinsic catalytic performance normalized on ECSA would be seriously underestimated.^73,84^ As shown in Figure S15, CV measurements evidence that the capacitance of SWCNTs obscures that of our catalytic films, thus impeding estimating the *C*_dl_ of our catalytic films. Although the *C*_dl_ of our catalytic films could be measured using flat substrates (as shown in Figure S1), the electrical contact between catalytic flakes and such substrates is not properly established, impeding a reliable quantitative ECSA analysis also in this case. Therefore, in agreement with literature recommendations,^82,84^ we evaluate the intrinsic activity of our electrocatalysts by specifically focusing on the mass activity of our electrodes at various potentials. Figure 7a and 7b shows the mass activity of our thermally treated electrodes at various overpotentials ranging from 0.15 to 0.3 V in 0.5 M H_2_SO_4_ and from 0.2 to 0.3 in 1 M KOH (within these ranges, artifacts arising from the presence of capacitive contribution at low current densities are negligible). In 0.5 M H_2_SO_4_, H-TaS_2_:H-TaSe_2_-Ar/H_2_@600°C displays a mass activity of the catalytic films of 114 and 314 A g^–1^ at overpotentials as low as 150 and 200 mV, respectively.

Table S3 reports a comparison between the geometric (η_10_) and intrinsic performance (mass activity) of our heterogeneous electrodes with those previously reported in the literature with similar materials (for which the production method is also specified).^39−41,43,44,85−92^ Our heterogeneous electrocatalysts reach performances significantly superior to MoS_2_-based catalysts^29,93^ and most of the group 5 TMDs reported in the literature, except for those produced by CVD methods and subsequently electrochemically treated with thousands of CV cycles^40^ or doped with noble-metal atoms.^87,94^ Therefore, our catalysts prove that it is possible to reach the performance predicted for group 5 TMDs with scalable and practical methods.

## 4 Conclusions

In summary, we produced single/few-layer flakes of H-TaS_2_ and H-TaSe_2_ through an eco-friendly liquid-phase exfoliation (LPE) of their crystals in 2-propanol. The as-produced flakes have been used in the form of films to catalyze the hydrogen evolution reaction (HER) in both acidic and alkaline media. More in detail, thermal treatment in a H_2_-rich atmosphere has been used to texturize the morphology of the catalytic films, increasing their porosity and the number of the HER active edges of the flakes. Our method has been compared to the prototypical electrochemical CV cycling process, as previously reported in the literature^40,41^ and licensed documents.^47^ As supported by ab initio molecular dynamic simulations and density functional theory calculations,^42^ the H-TaS_2_ flakes have been hybridized with the H-TaSe_2_ flakes to tune the Δ*G*_H_ of the H-TaS_2_ basal planes to the optimal thermo-neutral value in the resulting heterogeneous configurations. In 0.5 M H_2_SO_4_, the designed heterogeneous catalysts based on Ta dichalcogenides outperform their single counterparts, showing an overpotential at the cathodic current density of 10 mA cm^–2^ (η_10_) of 120 mV and high mass activity of 314 A g^–1^ at an overpotential of 200 mV. In 1 M KOH, they show a η_10_ of 230 mV and a mass activity of 220 A g^–1^ at an overpotential of 300 mV. Our heterogeneous electrodes show a durable HER activity over 12 h of nonstop operation at a fixed potential corresponding to a starting current density of −80 mA cm^–2^. Our results furnish new guidelines for the use of the metallic group 5 TMDs for the HER by means of scalable material preparation and electrode processing.

## Supporting Information Available

This material is available free of charge on the ACS Publication Web sites. The Supporting Information is available free of charge at https://pubs.acs.org/doi/10.1021/acscatal.9b03184.

Supplementary SEM-coupled EDS analysis of the 2H-TaS_2_ and 2H-TaSe_2_ crystals; double-layer capacitance measurements of the H-TaS_2_ films; TEM and SEM-coupled EDS analysis of the exfoliated H-TaSe_2_ sample; SEM analysis of the H-TaSe_2_ and H-TaS_2_:H-TaSe_2_ electrodes; electrochemical characterization of the H-TaSe_2_ electrodes; electrochemical stability of the heterogeneous H-TaS_2_:H-TaSe_2_ electrodes (PDF)
