# Supplementary material for: TaS2, TaSe2, and Their Heterogeneous Films as Catalysts for the Hydrogen Evolution Reaction
Source: ACS Catal. 2020 Feb 10;10(5):3313–25. doi: 10.1021/acscatal.9b03184 (PMC8016161; doi:10.1021/acscatal.9b03184)
Supplement: Supplementary file 1 — cs9b03184_si_001.pdf [file cs9b03184_si_001.pdf]

Supporting Information

TaS_2, $TaSe_2$ and Their Heterogeneous Films as Catalysts for Hydrogen Evolution Reaction

Leyla Najafi,[Φ,‡] *Sebastiano Bellani,*[Φ,‡] *Reinier Oropesa-Nuñez,*[□] *Beatriz Martín-García,*[Φ] *Mirko Prato,*[Δ] *Lea Pasquale,*[Δ] *Jaya-Kumar Panda,*[Φ] *Petr Marvan,*[Σ] *Sofer Zdeněk,*[Σ] *and Francesco Bonaccorso**[Φ,□]

[Φ] Graphene Labs, Istituto Italiano di Tecnologia, via Morego 30, 16163, Genova, Italy.

[□] BeDimensional Spa., via Albisola 121, 16163 Genova, Italy.

[Δ] Materials Characterization Facility, Istituto Italiano di Tecnologia, via Morego 30, 16163 Genova, Italy.

Department of Inorganic Chemistry, University of Chemistry and Technology Prague, Technická 5, 166 28 Prague 6, Czech Republic.

* Francesco Bonaccorso. Tel: +39 01071781795. E-mail: francesco.bonaccorso@iit.it

S1. Scanning electron microscopy-coupled energy dispersive X-ray spectroscopy analysis of the as-synthetized 2H-TaS_2 crystals

Table S1 reports the chemical composition of the as-synthetized 2H-TaS_2 crystals, as estimated by scanning electron microscopy (SEM)-coupled energy dispersive X-ray spectroscopy (EDS) analysis reported in the main text (**Figure 2a-c**). As discussed in the main text, SEM-coupled EDS analysis evidences a near-ideal stoichiometric phase of the 2H-TaS_2 crystals (S-to-Ta ratio = 1.9), in agreement with previous studies.[1,2] The significant atomic content of O and C is associated to the carbon tape used as the sample substrate, and is not attributed to impurities of the as-synthetized crystals (in agreement with complementary characterization shown in the manuscript).

Table S1. Elemental composition of the as-synthetized 2H-TaS_2 crystals, as estimated by the SEM-coupled EDS analysis

**Element**	**Atomic content (%)**
Ta	22.5
S	43.1
O	11.4
C	23.1

S2. Double-layer capacitance measurements of H-TaS_2 films

The double-layer capacitance (C_{dl}) of catalytic films deposited onto a glassy carbon (GC) substrate (catalyst mass loading = 0.1 mg cm^{-2}) was estimated by cyclic voltammetry (CV) measurements in a non-Faradaic region at different potential scan rate (ranging from 10 to 400 mV s^{-1}). **Figure S1a,b** show the CV curve of H-TaSe2 flakes films before and after thermal treatment in a H_2-rich environment at 600 °C (samples herein named H-TaS_2 and H-TaS_2 – Ar/H_2@600 °C, respectively). By plotting the difference between anodic and cathodic current densities ($\Delta j = (j_a - j_c)$) at 0.25 V *vs.* RHE as a function of the scan rate (SR) (**Figure S1c**), C_{dl} can be calculated by: $C_{dl} = d(\Delta j)/d2(SR)$. The calculated C_{dl} values are 0.18 mF cm^{-2} for H-TaS_2 and 0.25 mF cm^{-2} for H-TaS_2 – Ar/H_2@600 °C. Therefore, C_{dl} of H-TaS_2 – Ar/H_2@600 °C increases by 39% compared to C_{dl} of as-produced H-TaS_2. Since C_{dl} is proportional to the electrochemically accessible surface area, these result indicate an increase of the porosity of the thermally treated catalytic films, allowing for an optimal electrolyte ion accessibility.

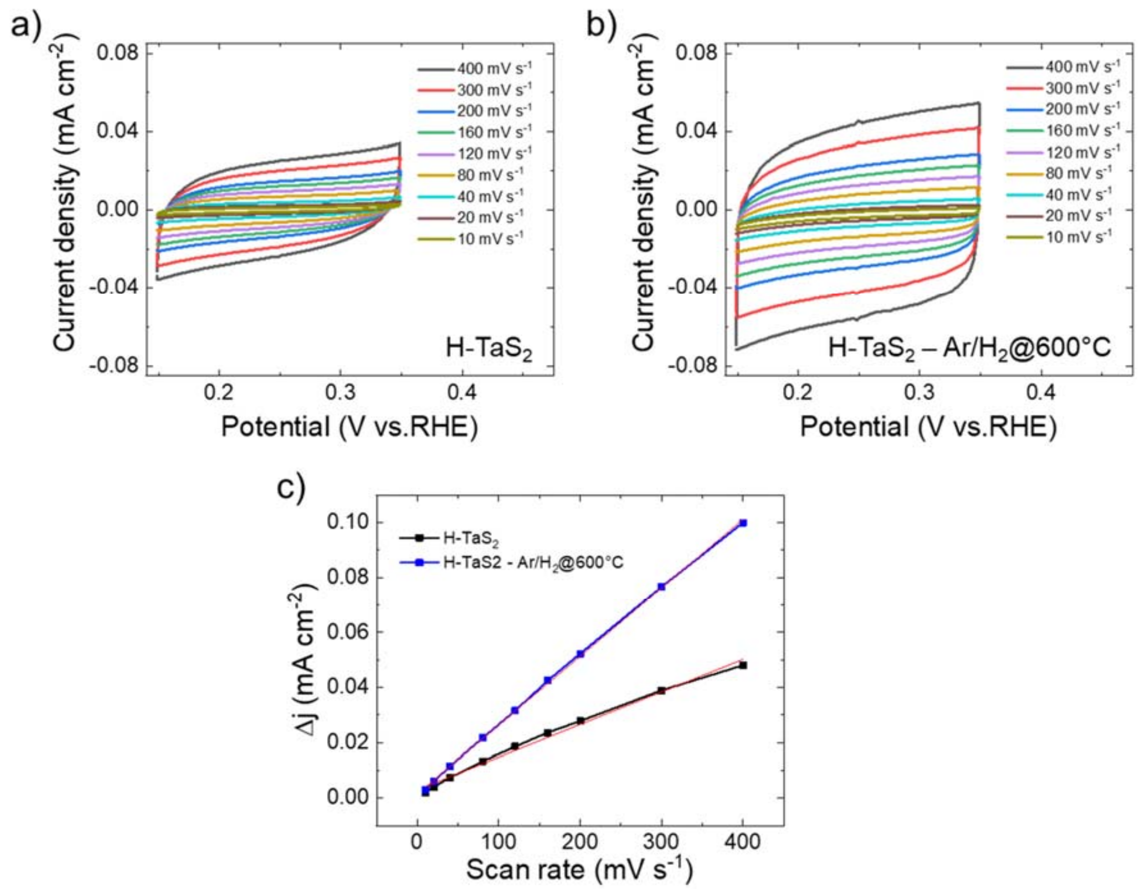

Figure S1. CV measurements at various potential SRs for the following catalytic films: a) as-produced H-TaS_2 films deposited onto glassy carbon (H-TaS_2); b) H-TaS_2 films deposited onto GC after thermal treatment in a H_2-rich environment at 600 °C (H-TaS_2 – Ar/H_2@600 °C). c) SR dependence of Δj for H-TaS_2 and H-TaS_2 – Ar/H_2@600 °C. The linear fit of the curves are also shown.

S3. X-ray diffraction measurements of H-TaS_2 films before and after thermal treatment

Figure S2 shows the X-ray diffraction spectra (XRD) spectra of a H-TaS_2 catalytic film on a glass substrate before and after thermal treatment at 600°C in H_2-rich environment (sample herein named H-TaS_2 and H-TaS_2 – Ar/H_2@600°C, respectively). The data reveal that the loss of chalcogens occurring during the thermal treatment (see details in main text, **Figure 3**), leads to the formation of elemental Ta, which subsequently undergoes to an oxidation when material is exposed to air. Since the flakes preserve their two-dimensional (2D) morphology (see SEM analysis in the main text, **Figure 3**) without evolving towards cluster-like structures formed by metal coalescence, the oxidation of H-TaS_2 flakes can be mainly ascribed to the surface of the material in contact with H_2-rich atmosphere.

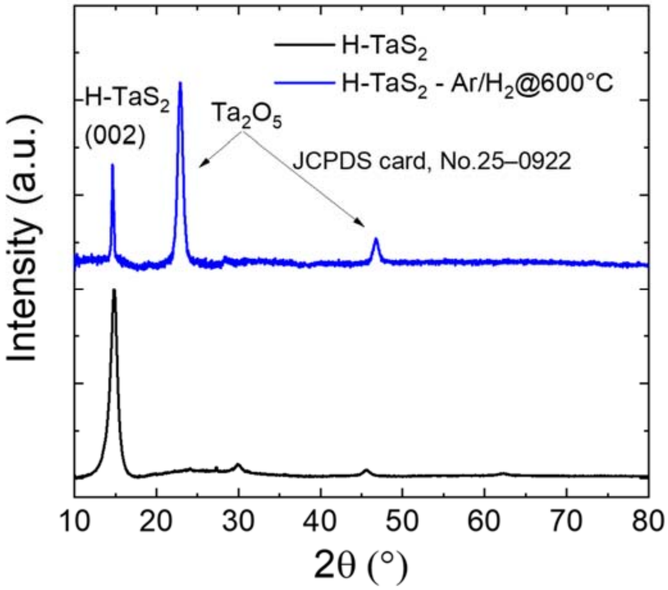

Figure S2. XRD spectra of H-TaS_2 and H-TaS_2 – Ar/H_2@600°C.

S4. Scanning electron microscopy-coupled energy dispersive X-ray spectroscopy analysis of as-synthetized 2H-$TaSe_2$ crystals

The as-produced 2H-$TaSe_2$ crystals were characterized by SEM-coupled EDS measurements (**Figure S3a-c**). Their analysis evidences a near-ideal stoichiometric phase (Se-to-Ta ratio = 2.2) (**Table S2**), in agreement with previous studies.[1,2] The excess of Se could be ascribed to one dimensional (1D)-like trigonal Se by-products of the synthesis of 2H-$TaSe_2$ crystals. These by-products can be formed by the recrystallization of polycrystalline Se.[3–5] The layered structure of 2H-$TaSe_2$ crystals is clearly visible on their edges, as shown by high-magnification SEM imaging (**Figure S3d)**. The significant atomic content of O and C is associated to the carbon tape used as the sample substrate, and is not attributed to impurities of the as-synthetized crystals (in agreement with complementary characterization shown in the manuscript).

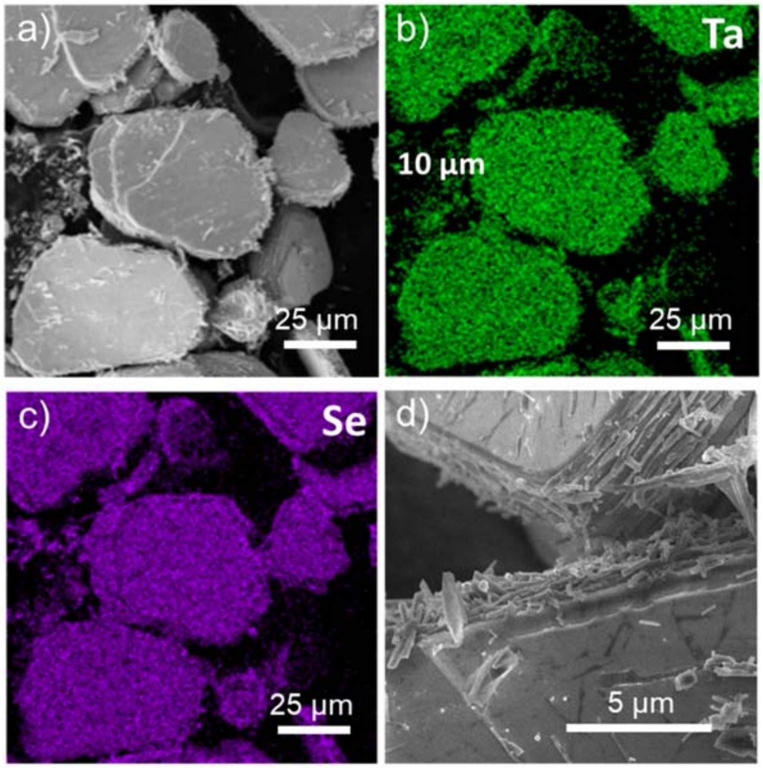

Figure S3. a) SEM image of the as-synthetized 2H-$TaSe_2$ crystals with corresponding EDS maps for b) the Ta (green) and c) the Se (violet). d) High-magnification SEM image of 2H-$TaSe_2$ crystal edges, showing their layered structure.

Table S2. Elemental composition of the as-synthetized 2H-TaS_2 crystals, as estimated by SEM-coupled EDS analysis.

Element	Atomic content (%)
Ta	8.2
Se	18.1
O	11.9
C	61.8

S5. Transmission electron microscopy measurements of the exfoliated H-$TaSe_2$ sample

Figure S4 reports a transmission electron microscopy (TEM) image of the exfoliated H-$TaSe_2$ sample, which consists of flakes and one-dimensional (1D) structures. As shown in the next section by SEM-coupled EDS analysis (**Figure S5**), the flakes correspond to the chemical structure of H-$TaSe_2$, while 1D structures are attributed to trigonal Se by-products, which are formed from dissolution-recrystallization mechanisms involving polycrystalline Se.[3–5]

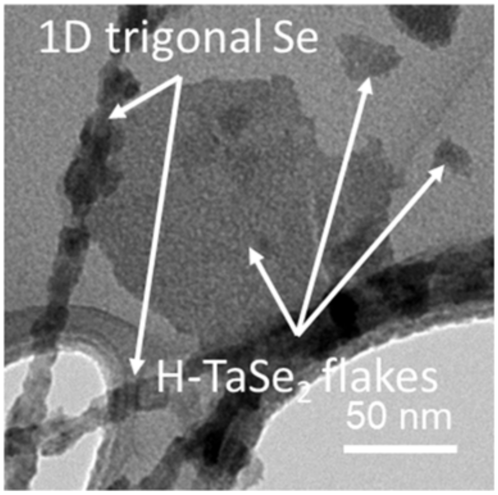

Figure S4. TEM image of the exfoliated H-$TaSe_2$ sample.

S6. Scanning electron microscopy-coupled energy dispersive X-ray spectroscopy analysis of the exfoliated H-$TaSe_2$ sample

Figure S5a-c report the SEM-coupled EDS analysis of H-$TaSe_2$ sample. Its SEM image (**Figure S5a**) shows that the samples consists of both flakes and 1D structures, in agreement with TEM analysis reported in the previous section (**Figure S4**). Elemental mapping (**Figure S5b-d**) clearly indicates that the flakes refer to the chemical structure of H-$TaSe_2$, while 1D structures are attributed to trigonal Se by-products, which are formed from dissolution-recrystallization mechanism involving polycrystalline Se.[3–5] These attributions are further confirmed by SEM-coupled EDS analysis focused on solely 1D structures (**Figure S5e-h**) or flakes (**Figure S5i-j**).

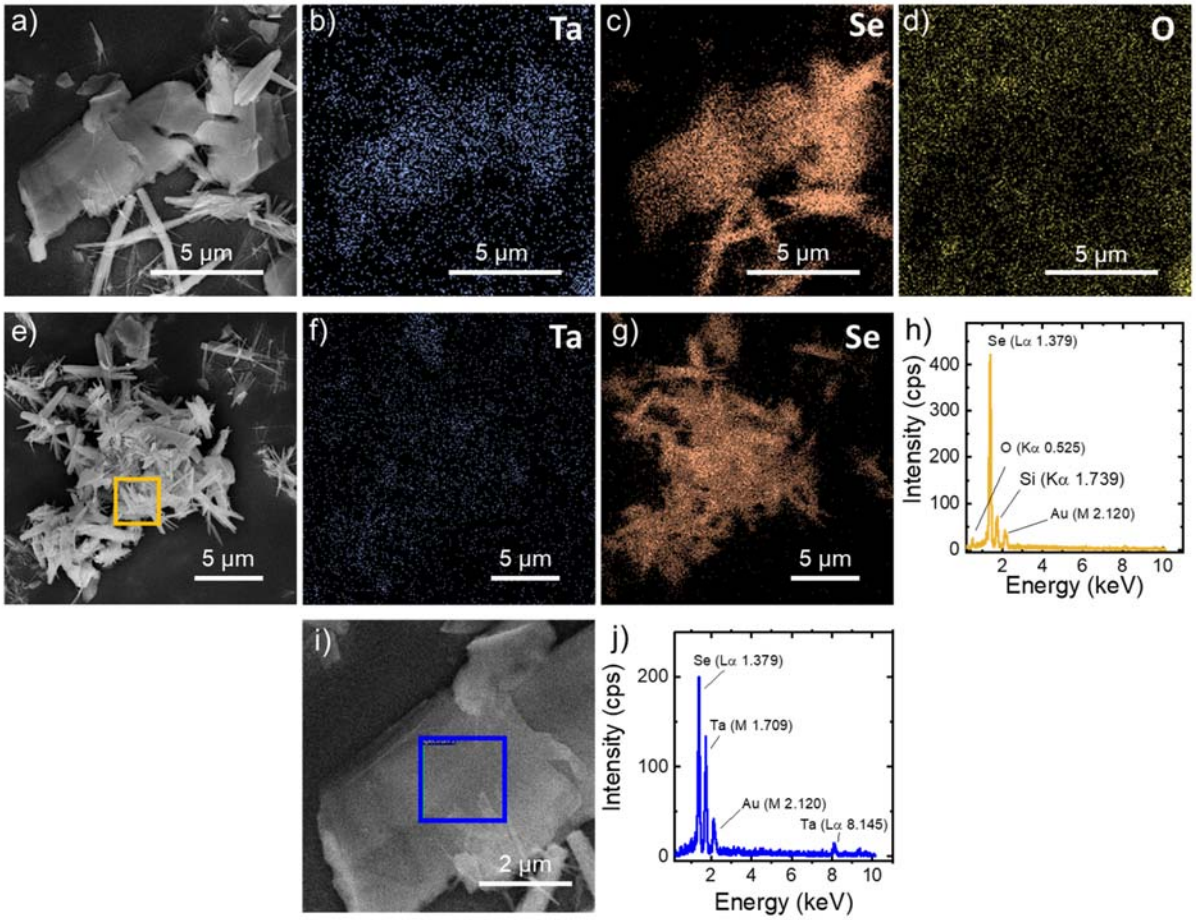

Figure S5. a) SEM image of the exfoliated H-$TaSe_2$ sample, consisting of both flakes and 1D structures, and corresponding EDS maps for b) the Ta (Lα = 8.14 keV, in blue) c) the Se (Lα = 1,38 keV, in orange) and d) the O (Kα = 0.52 keV, in yellow). The EDS mapping attributes the flakes to the chemical structure of H-TaS_2, while 1D structures are assigned to trigonal Se crystals. e) SEM image of 1D structures in the exfoliated H-$TaSe_2$ sample, and its EDS maps for f) the Ta (Lα = 8.14

keV, in blue) g) the Se (Lα = 1,38 keV, in orange). h) EDS spectrum corresponding to the images of panels e-g. i) SEM image of flakes in the exfoliated H-$TaSe_2$ sample and j) its EDS spectrum.

S7. Scanning electron microscopy measurements of H-$TaSe_2$ and H-TaS_2:H-$TaSe_2$ electrodes

Figure S6a,b report the the top-view SEM images of the H-$TaSe_2$ and the H-$TaSe_2$:H-TaS_2 electrodes, respectively. The as-produced electrodes display a wrinkled structure, which is different from the laminar one shown by H-TaS_2 electrodes. In fact, the 1D structures composing the exfoliated H-$TaSe_2$ samples partially impede a preferential in-plane arrangement of the flakes during their film deposition.

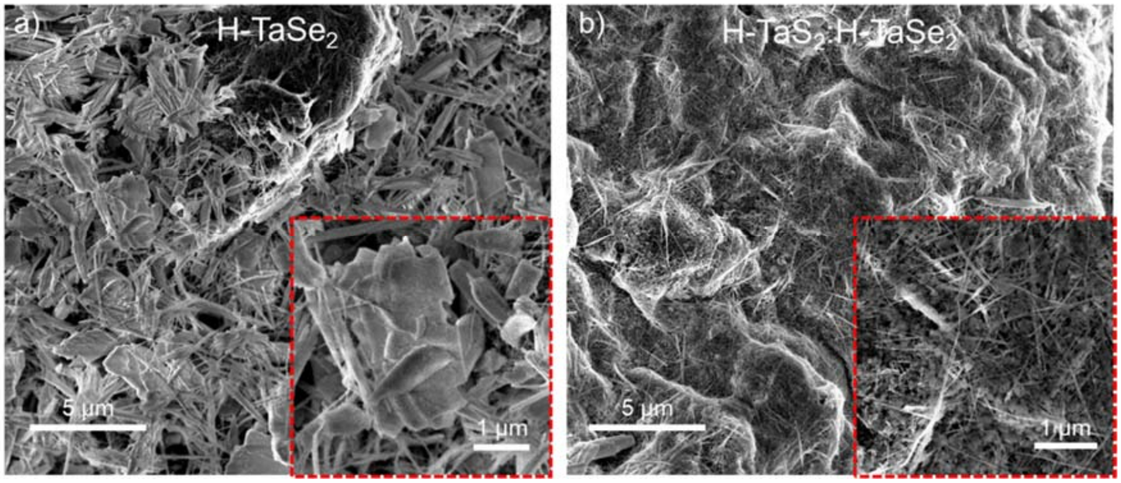

Figure S6. a,b) Top-view SEM images of H-$TaSe_2$ and H-$TaSe_2$:H-TaS_2 electrodes, respectively. The inset panels show SEM images of the same electrodes with a magnification higher than that of the main panels.

S8. Scanning electron microscopy-coupled energy dispersive X-ray spectroscopy analysis of heterogeneous H-TaS_2:H-$TaSe_2$ electrodes

Figure S7a-d report the top-view SEM-coupled EDS analysis of a representative H-TaS_2:H-$TaSe_2$ electrode. **Fig. S7e-h** show the cross-sectional SEM-coupled EDS analysis of the same H-TaS_2:H-$TaSe_2$ electrode. In both cases, the data show a homogeneous distribution of both S and Se elements, suggesting the absence of single material domains.

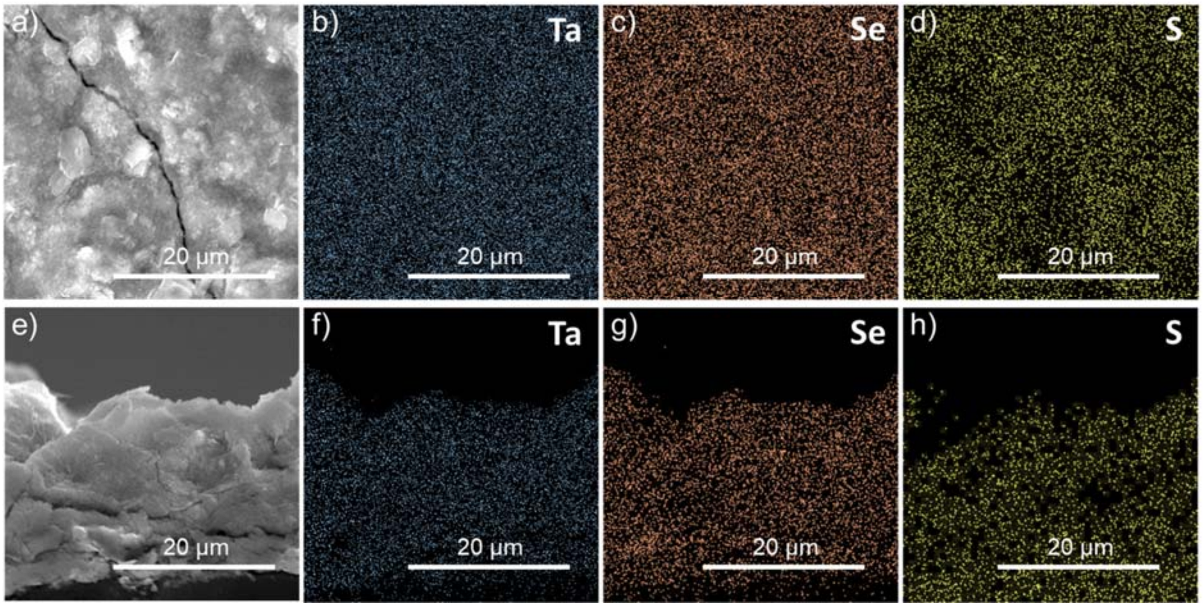

Figure S7. a,d) Top-view SEM images of H-TaS_2:H-$TaSe_2$ electrodes, with the corresponding elemental maps for Ta (Lα = 8.14 keV, in blue), Se (Kα = 11.2 keV, in orange) and S (Kα = 2.3 keV, in yellow). e,f) Cross-section SEM images of the H-TaS_2:H-$TaSe_2$ electrodes, with the corresponding elemental maps for Ta (Lα = 8.14 keV, in blue), Se (Kα = 11.2 keV, in orange) and S (Kα = 2.3 keV, in yellow).

S9. X-ray diffraction measurement of H-$TaSe_2$ films before and after thermal treatment

Figure S8 shows the XRD spectra of H-$TaSe_2$ films on glass substrates before and after thermal treatment at 600°C in H_2-rich environment (sample herein named H-$TaSe_2$ and H-$TaSe_2$ – Ar/H_2@600°C, respectively). The data evidence that the loss of chalcogens occurring during the thermal treatment (see details in the main text, **Figure 3**) leads to the formation of elemental Ta, which subsequently undergoes to an oxidation when the catalytic film is exposed to air. Similar effects have been observed and discussed for the case of H-TaS_2 catalytic films (see Section S3).

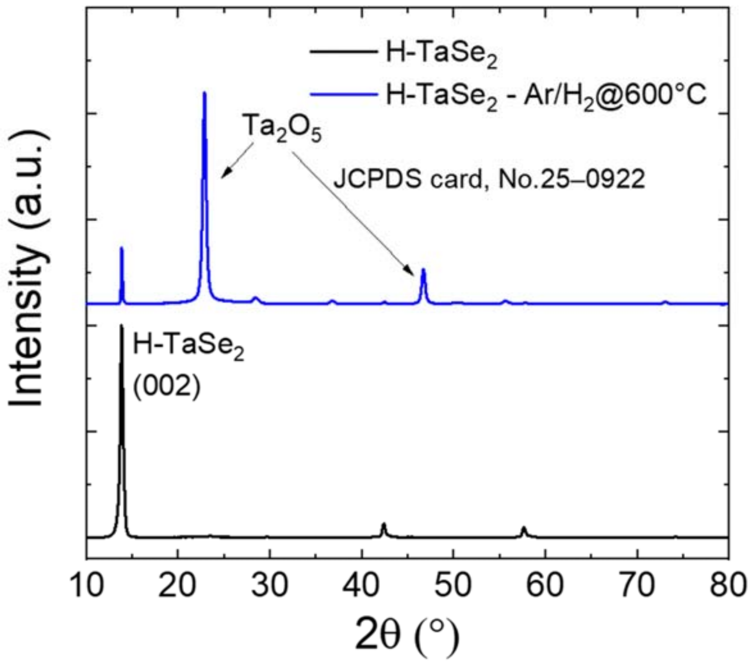

Figure S8. XRD spectra of $TaSe_2$ and H-$TaSe_2$ – Ar/H_2@600°C.

S10. Electrochemical characterization of H-$TaSe_2$ electrodes

Figure S9a,b show the *iR*-corrected linear sweep voltammetry (LSV) curves in 0.5 M H_2SO_4 and 1 M KOH, respectively, measured for the electrodes before and after the thermal treatment (samples named H-$TaSe_2$ and H-$TaSe_2$ – Ar/H_2@600°C, respectively). In addition, the LSV curves measured for the untreated electrode after 1000 CV cycles (sample named H-$TaSe_2$ – CV@1000 cycles), the commercial Pt/C (benchmark) and the SWCTNs (catalyst support) are also plotted. In 0.5 M H_2SO_4, H-$TaSe_2$ – Ar/H_2@600°C exhibits a HER-activity significantly higher than the one of the as-produced electrodes (H-$TaSe_2$). In particular, H-$TaSe_2$ – Ar/H_2@600°C shows a η_{10} of 200 mV, which is slightly superior to that of H-$TaSe_2$ – CV@1000 cycles (η_{10} of 210 mV). Similar results were also measured in 1 M KOH, in which H-$TaSe_2$ – Ar/H_2@600°C shows a η_{10} of 260 mV, whereas the as-produced H-$TaSe_2$ and the H-$TaSe_2$ – CV@1000 cycles electrodes display η_{10} of 490 mV and 360 mV, respectively.

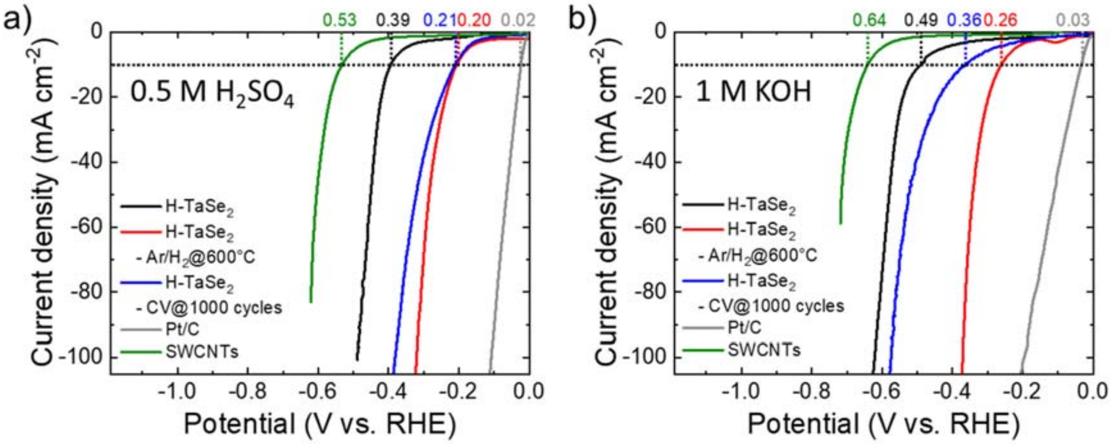

Figure S9. a,b) *iR*-corrected LSV curves measured for H-$TaSe_2$, H-$TaSe_2$ – Ar/H_2@600°C and H-$TaSe_2$ – CV@1000 cycles in 0.5 M H_2SO_4 and 1 M KOH, respectively. The LSV curves measured for Pt/C (benchmark) and SWCTNs (catalyst support) are also shown for comparison. The η_{10} values measured for the various electrodes are also shown.

S11. Electrochemical stability tests of heterogeneous H-TaS$_2$:H-TaSe$_2$ electrodes

Figure S10 shows the chronoamperometry measurements for the thermally treated heterogeneous electrodes (named (H-TaS$_2$:H-TaSe$_2$ – Ar/H$_2$@600°C in the main text) at a fixed potential corresponding to an initial cathodic current density of 80 mA cm^{-2}. In 0.5 M H_2SO_4, the electrode retains 97% of the initial current density after 12 h, thus promising a durable HER-activity. In alkaline condition, the electrode degrades during the first 4 h, thenceforth its current density is progressively stabilized (current density equal to 81% of the initial one after 12 h).

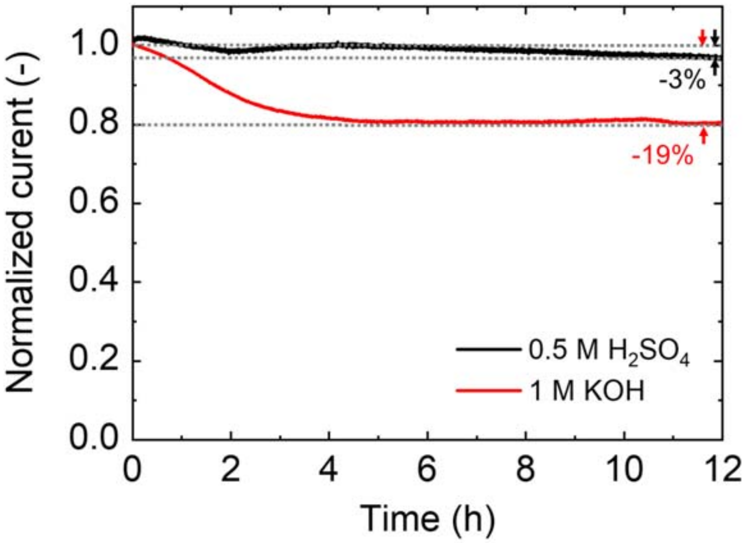

Figure S10. Chronoamperometry measurements (j-t curves) at a fixed potential corresponding to an initial cathodic current of 80 mA cm^{-2} for H-TaS$_2$:H-TaSe$_2$ – Ar/H$_2$@600°C electrodes in acidic (0.5 M H_2SO_4) and alkaline (1 M KOH) solutions. The percentage current density degradation after 12 h is also indicated in the plot.

S12. Scanning electron microscopy analysis of H-TaS_2 electrodes before and after cyclic voltammetry cycling

Figure S11 reports the image of the untreated and the thermally treated H-TaS_2 electrodes (*i.e.*, H-TaS_2 and H-TaS_2 – Ar/H_2@600°C) before and after the CV cycling (1000 cycles). **Figure S11a,b** show that H-TaS_2 significantly changes its morphology after CV cycling, resulting in a fragmented surface, in agreement with previous studies on H-TaS_2 electrodes.[6–8] Differently, H-TaS_2 – Ar/H_2@600°C does not show any significant morphology change before and after CV cycling (**Figure S11c,d**). This indicates that the initial porosity of the thermo-texturized electrode is enough to allow the evolved H_2 to escape from the electrode surface without altering its morphology.

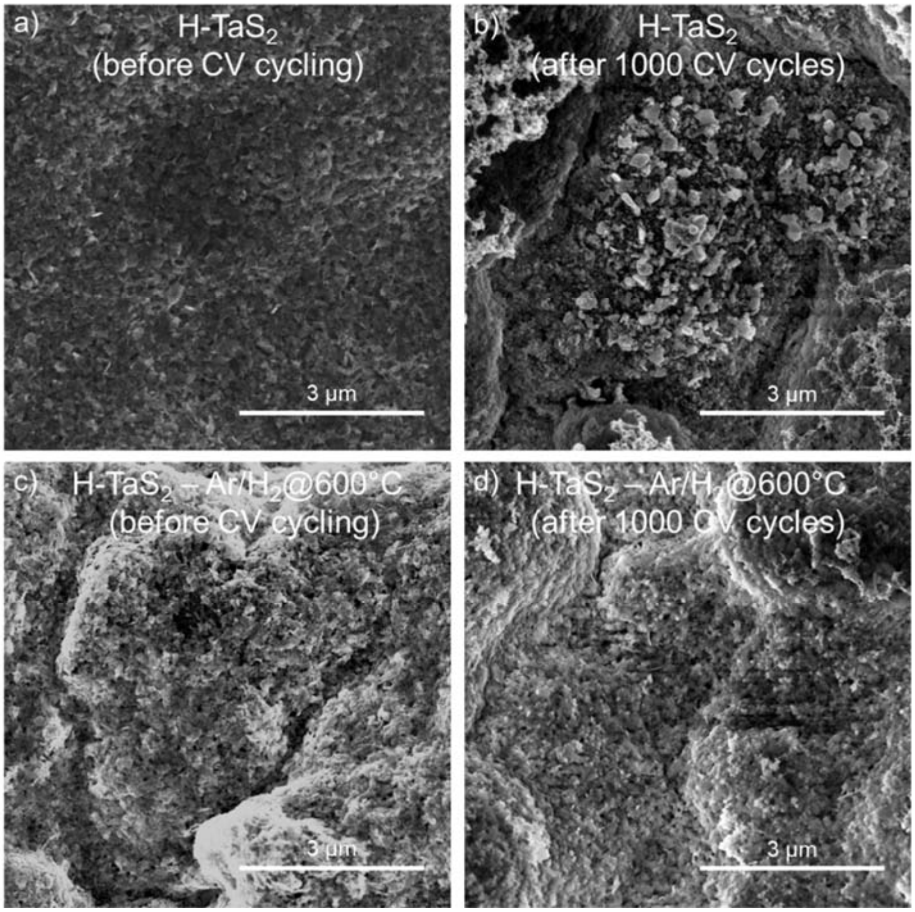

Figure S11. a,b) SEM images of H-TaS_2 electrode before and after 1000 CV cycles. c,d) SEM images of H-TaS_2 – Ar/H_2@600°C electrode before and after 1000 CV cycles.

S13. Morphological and spectroscopic characterization of H-TaS_2:H-$TaSe_2$ – CV@1000 cycles

Figure S12 reports the top-view SEM-EDS analysis of H-TaS_2:H-$TaSe_2$ – CV@1000 cycles. In comparison with non-thermally electrode (H-TaS_2:H-$TaSe_2$, see analysis in **Figure S7**), this electrode shows significant differences in its surface morphology, which appear more corrugated. This result confirms those observed in **Figure S11** for H-TaS_2 and H-TaS_2 – CV@1000 cycles. In addition, EDS maps show a chemical redistribution of S, which indicates the possibility of chemical changes at the surface of the catalytic films.

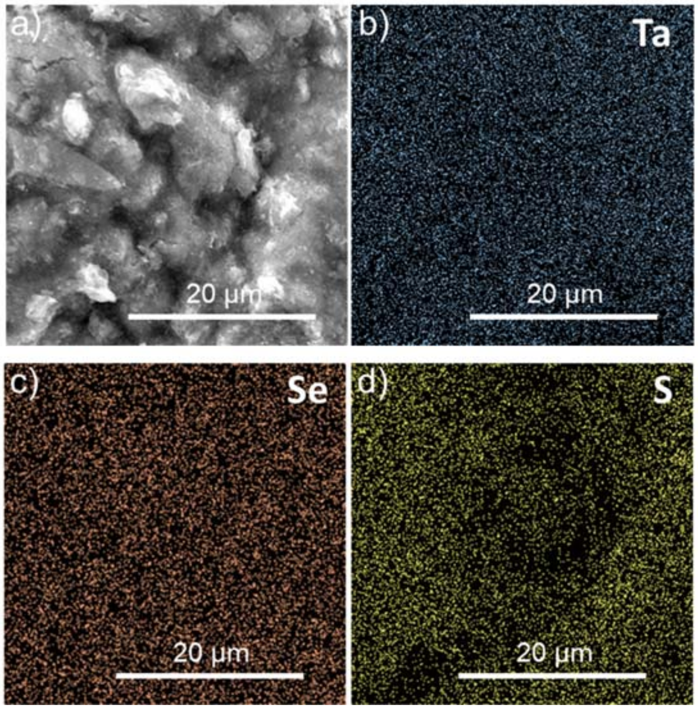

Figure S12. a) Top-view SEM images of H-TaS_2:H-$TaSe_2$ – CV@1000 cycles, with its EDS maps for b) Ta (Lα = 8.14keV, in blue), c) Se (Kα = 11.2 keV, in orange) and d) S (Kα = 2.3 keV, in yellow).

As shown in **Figure S13**, XRD measurements of H-TaS_2:H-$TaSe_2$ – CV@1000 cycles further evidence chemical changes on its surface. In particular, after the electrochemical treatment, the intensity of the XRD peaks attributed to the oxides (namely Ta_2O_5) increases relatively to the peaks of H-TaS_2 and H-$TaSe_2$.

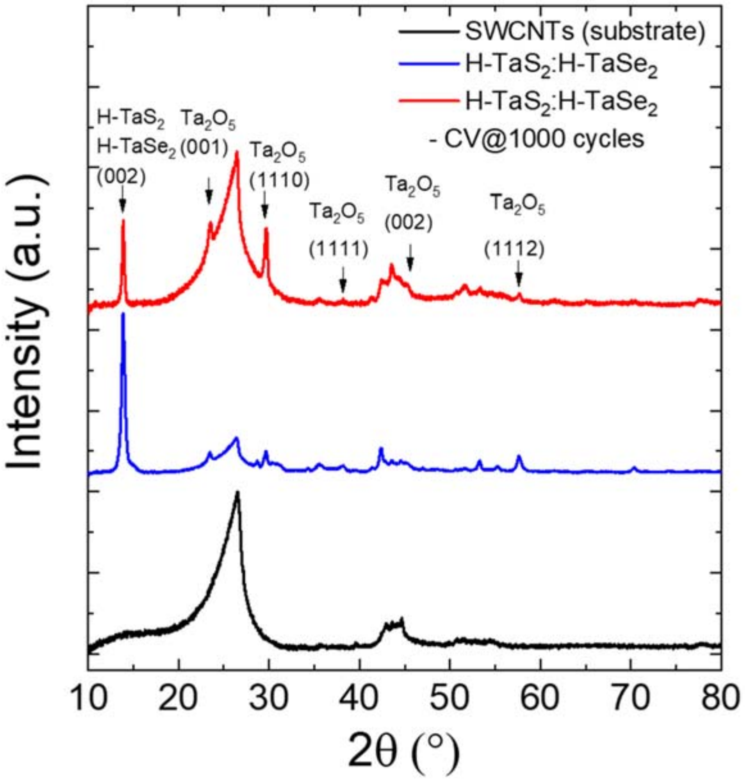

Figure S13. XRD spectra of SWCNTs (substrate), $H\text{-}TaS_2$:$H\text{-}TaSe_2$ and $H\text{-}TaS_2$:$H\text{-}TaSe_2$ – CV@1000 cycles.

These results partially contradict those previously reported for $H\text{-}TaS_2$ electrodes in literature, where it is claimed that $H\text{-}TaS_2$ catalysts preserve their chemical integrity.[6–8] At current stage, more specific studies on electrodes with catalyst mass loadings similar to those of our electrodes are still needed to provide definitive understandings regarding to possible chemical changes of these catalysts during HER.

S14. Electrochemical stability tests in alkaline condition using polytetrafluoroethylene cell

The dissolution of the quartz of the cell in alkaline media could alter the electrolyte composition, affecting the HER-activity of the electrodes. In order to exclude these effects, the stability of the heterogeneous electrode was also tested in an alkaline resistant polytetrafluoroethylene (PTFE) cell. As shown in **Figure S14**, the data confirm an initial degradation of the electrodes (-8% after 1.9 h). Subsequently, the HER-activity of the electrode progressively increases over time (+16% after 12 h), suggesting an evolution toward a progressive electrochemical equilibrium, which was also observed in the quartz cell. Interestingly, our durable HER-activity was achieved without using any binder, such as Nafion, which could prospectively improve the mechanical stability of the electrodes during HER. In fact, the mechanical stresses originated by the gas evolution have been demonstrated to be the cause of the self-optimizing fragmentation of the initial catalytic group-5 TMDs.[6-8] However, these effects could cause significant material losses, that have to be controlled for practical applications.

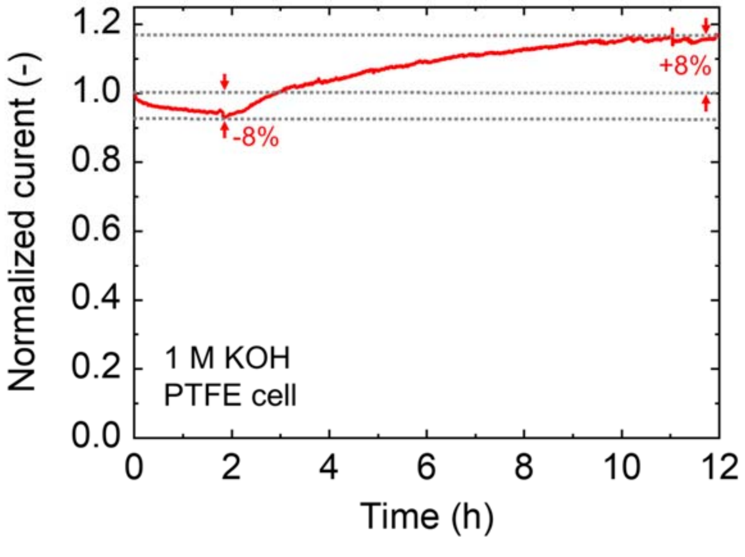

Figure S14. Chronoamperometry measurements at a fixed potential corresponding to an initial cathodic current of 80 mA cm^{-2} for H-TaS_2:H-$TaSe_2$ – Ar/H_2@600°C electrode in 1 M KOH solutions contained in an alkaline resistant PTFE cell. The percentage current density degradation after 12 h is also indicated in the plot.

S15. Double-layer capacitance measurements of the investigated electrodes

Figure S15a-c reports the CV measurements of SWCNTs (buckypaper) and two of the investigated electrodes based on H-TaS_2 and H-$TaSe_2$ catalytic films deposited on SWCNTs (sample named H-TaS_2 and H-$TaSe_2$, respectively). As previously shown in Section 2, by plotting Δj at 0.275 V vs. RHE as a function of SR (**Figure S15d**), the C_{dl} can be calculated by: $C_{dl} = d(\Delta j)/d2(SR)$. By considering SR ≤ 80 mV s^{-1}, the resistive loss can be neglected and the C_{dl} can be calculated for all the investigated electrodes. All the electrodes show a similar C_{dl} (around 500 mF cm^{-2}), indicating that the C_{dl} of the SWNCTs obscure the one of the catalytic films.

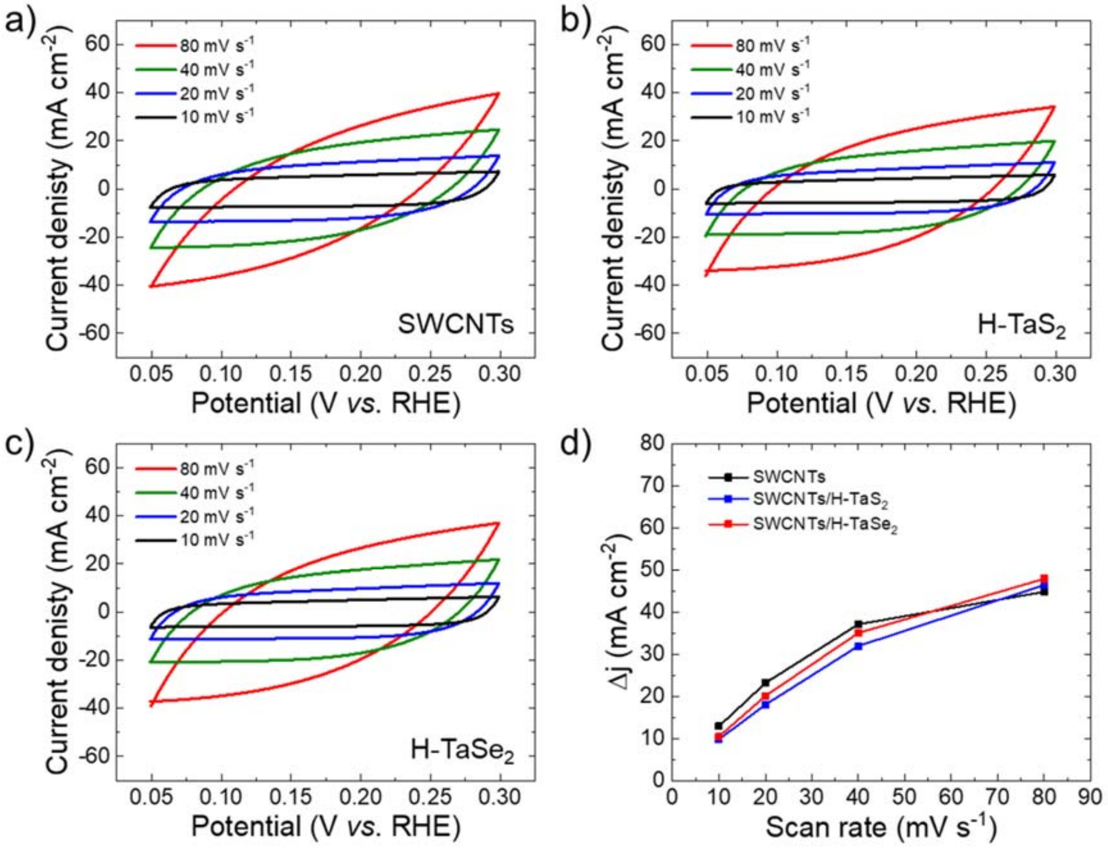

Figure S15. CV measurements at various potential SRs for: a) SWCNTs (buckpaper) ; b) H-TaS_2 and c) H-$TaSe_2$ films deposited onto SWCNTs (sample named H-TaS_2 and SWCNTs/H-$TaSe_2$, respectively). c) SR dependence of the Δj for the various electrodes.

S16. Comparison between the performance of our catalytic materials and those reported for similar materials in literature

Table 1. HER-performance of our heterogeneous catalyst in 0.5 M H_2SO_4 in comparison with those reported for similar materials (group-V TMDs) in literature.

**Catalyst**	**Production methods**	**η_{10} (mV)**	**Mass activity (A g^{-1})**	**Reference**
H-TaS_2:H-TaS_2 – Ar/H_2@600°C	LPE + thermal treatment in H_2-rich environment	120	114@150mV 314@200mV	this work
2H-TaS_2/Au	CVD and 5000 CV cycles	65	N.D.	[7]
H-TaS_2	CVD and 5000 CV ccles	60	~2910 @150mV	[6]
2H-TaS_2	Solid-state reaction + hydrothermal method	145	N.D.	[9]
2H-TaS_2	Arc discharge method	> 350	60-70 @400mV	[10]
3R-TaS_2	Direct thermal synthesys	196	N.D.	[11]
2H-TaS_2	CVD	101	N.D.	[12]
2H-TaS2	LPE + plasma etching	564	~42@564mV	[13]
1T-TaS2	CVD	205	N.D.	[14]
$Pd_{0.1}TaS_2$	Chemical reduction method	241	400@241 mV	[15]
3R-NbS_2	Chemical solid reaction and 20000 CV cycles	58	N.D.	[16]
3R-NbS_2	ambient pressure annealing	0.5	N.D.	[17]
2H-NbS_2	Electrochemical exfoliation	200-250	43.15@250mV	[18]
2H-NbS_2:2H-$MoSe_2$	LPE	100	50@100mV	[19]
Pd_xNbS_2	Direct synthesis	157	1470@200mV	[20]
1T-MoS_2	Chemical intercalation	~200	~20@150mv	[21]
2H-MoS_2 (double gyroid MoS_2)	Electrodeposition into the double-gyroid silica template	~280	~16@150mV	[22]

REFERENCES

(1) Luxa, J.; Mazánek, V.; Pumera, M.; Lazar, P.; Sedmidubský, D.; Callisti, M.; Polcar, T.; Sofer, Z. 2H→1T Phase Engineering of Layered Tantalum Disulfides in Electrocatalysis: Oxygen Reduction Reaction. *Chem. – A Eur. J.* **2017**, *23*, 8082–8091.

(2) Chia, X.; Ambrosi, A.; Lazar, P.; Sofer, Z.; Pumera, M. Electrocatalysis of Layered Group 5 Metallic Transition Metal Dichalcogenides (MX2, M = V, Nb, and Ta; X = S, Se, and Te). *J. Mater. Chem. A* **2016**, *4*, 14241–14253.

(3) Murphy, K. E.; Altman, M. B.; Wunderlich, B. The Monoclinic-to-trigonal Transformation in Selenium. *J. Appl. Phys.* **1977**, *48*, 4122–4131.

(4) Cheng, B.; Samulski, E. T. Rapid, High Yield, Solution-Mediated Transformation of Polycrystalline Selenium Powder into Single-Crystal Nanowires. *Chem. Commun.* **2003**, 16, 2024–2025.

(5) Lu, J.; Xie, Y.; Xu, F.; Zhu, L. Study of the Dissolution Behavior of Selenium and Tellurium in Different Solvents - A Novel Route to Se, Te Tubular Bulk Single Crystals. *J. Mater. Chem.* **2002**, *12*, 2755–2761.

(6) Liu, Y.; Wu, J.; Hackenberg, K. P.; Zhang, J.; Wang, Y. M.; Yang, Y.; Keyshar, K.; Gu, J.; Ogitsu, T.; Vajtai, R.; Lou, J.; Ajayan, P. M.; Wood, B. C.; Yakobson, B. I. Self-Optimizing, Highly Surface-Active Layered Metal Dichalcogenide Catalysts for Hydrogen Evolution. *Nat. Energy* **2017**, *2*, 17127.

(7) Shi, J.; Wang, X.; Zhang, S.; Xiao, L.; Huan, Y.; Gong, Y.; Zhang, Z.; Li, Y.; Zhou, X.; Hong, M.; Fang, Q.; Zhang, Q.; Liu, X.; Gu, L.; Liu, Z.; Zhang, Y. Two-Dimensional Metallic Tantalum Disulfide as a Hydrogen Evolution Catalyst. *Nat. Commun.* **2017**, *8*, 958.

(8) Hackenberg, K.; Keyshar, K.; Wu, J.; Liu, Y.; Ajayan, P.; Wood, B.; Yakobson, B. Self-Improving Electrocatalysists for Gas Evolution Reactions. US 2016/0153098 A1, 2016.

(9) Zhang, M.; He, Y.; Yan, D.; Xu, H.; Wang, A.; Chen, Z.; Wang, S.; Luo, H.; Yan, K. Multifunctional 2H-TaS2 Nanoflakes for Efficient Supercapacitors and Electrocatalytic

Evolution of Hydrogen and Oxygen. *Nanoscale* **2019**, *11*, 22255–22260.

(10) Raj, I.; Duan, Y.; Kigen, D.; Yang, W.; Hou, L.; Yang, F.; Li, Y. Catalytically Enhanced Thin and Uniform TaS2 Nanosheets for Hydrogen Evolution Reaction. *Front. Mater. Sci.* **2018**, *12*, 239–246.

(11) Feng, Y.; Gong, S.; Du, E.; Chen, X.; Qi, R.; Yu, K.; Zhu, Z. 3R TaS2 Surpasses the Corresponding 1T and 2H Phases for the Hydrogen Evolution Reaction. *J. Phys. Chem. C* **2018**, *122*, 2382–2390.

(12) Yu, Q.; Luo, Y.; Qiu, S.; Li, Q.; Cai, Z.; Zhang, Z.; Liu, J.; Sun, C.; Liu, B. Tuning the Hydrogen Evolution Performance of Metallic 2D Tantalum Disulfide by Interfacial Engineering. *ACS Nano* **2019**, *13*, 11874–11881.

(13) Li, H.; Tan, Y.; Liu, P.; Guo, C.; Luo, M.; Han, J.; Lin, T.; Huang, F.; Chen, M. Atomic-Sized Pores Enhanced Electrocatalysis of TaS2 Nanosheets for Hydrogen Evolution. *Adv. Mater.* **2016**, *28*, 8945–8949.

(14) Huan, Y.; Shi, J.; Zou, X.; Gong, Y.; Zhang, Z.; Li, M.; Zhao, L.; Xu, R.; Jiang, S.; Zhou, X.; Hong, M.; Xie, C.; Li, H.; Lang, X.; Zhang, Q.; Gu, L.; Yan, X.; Zhang, Y. Vertical 1T-TaS2 Synthesis on Nanoporous Gold for High-Performance Electrocatalytic Applications. *Adv. Mater.* **2018**, *30*, 1705916.

(15) Wang, D.; Wang, X.; Lu, Y.; Song, C.; Pan, J.; Li, C.; Sui, M.; Zhao, W.; Huang, F. Atom-Scale Dispersed Palladium in a Conductive Pd0.1TaS2 Lattice with a Unique Electronic Structure for Efficient Hydrogen Evolution. *J. Mater. Chem. A* **2017**, *5*, 22618–22624.

(16) Zhang, J.; Wu, J.; Zou, X.; Hackenberg, K.; Zhou, W.; Chen, W.; Yuan, J.; Keyshar, K.; Gupta, G.; Mohite, A.; Ajayan, P. M.; Lou, J. Discovering Superior Basal Plane Active Two-Dimensional Catalysts for Hydrogen Evolution. *Mater. Today* **2019**, *25*, 28–34.

(17) Gopalakrishnan, D.; Lee, A.; Thangavel, N. K.; Reddy Arava, L. M. Facile Synthesis of Electrocatalytically Active NbS2 Nanoflakes for an Enhanced Hydrogen Evolution Reaction (HER). *Sustain. Energy Fuels* **2018**, *2*, 96–102.

(18) Si, J.; Zheng, Q.; Chen, H.; Lei, C.; Suo, Y.; Yang, B.; Zhang, Z.; Li, Z.; Lei, L.; Hou, Y.; Ostrikov, K. (Ken) Scalable Production of Few-Layer Niobium Disulfide Nanosheets via Electrochemical Exfoliation for Energy-Efficient Hydrogen Evolution Reaction. *ACS Appl. Mater. Interfaces* **2019**, *11*, 13205–13213.

(19) Najafi, L.; Bellani, S.; Oropesa-Nuñez, R.; Martín-García, B.; Prato, M.; Mazánek, V.; Debellis, D.; Lauciello, S.; Brescia, R.; Sofer, Z.; Bonaccorso, F. Niobium Disulphide (NbS2)-Based (Heterogeneous) Electrocatalysts for an Efficient Hydrogen Evolution Reaction. *J. Mater. Chem. A* **2019**, *7*, 25593–25608.

(20) Huang, C.; Wang, X.; Wang, D.; Zhao, W.; Bu, K.; Xu, J.; Huang, X.; Bi, Q.; Huang, J.; Huang, F. Atomic-Pillar Effect in PdxNbS2 to Boost Basal-Plane Activity for Stable Hydrogen Evolution. *Chem. Mater.* **2019**, *31*, 4726–4731.

(21) Voiry, D.; Salehi, M.; Silva, R.; Fujita, T.; Chen, M.; Asefa, T.; Shenoy, V. B.; Eda, G.; Chhowalla, M. Conducting MoS2 Nanosheets as Catalysts for Hydrogen Evolution Reaction. *Nano Lett.* **2013**, *13*, 6222–6227.

(22) Kibsgaard, J.; Chen, Z.; Reinecke, B. N.; Jaramillo, T. F. Engineering the Surface Structure of MoS2 to Preferentially Expose Active Edge Sites for Electrocatalysis. *Nat. Mater.* **2012**, *11*, 963.
